# Prevention of Dominant IgG Adsorption on Nanocarriers in IgG‐Enriched Blood Plasma by Clusterin Precoating

**DOI:** 10.1002/advs.201802199

**Published:** 2019-04-04

**Authors:** Domenik Prozeller, Jorge Pereira, Johanna Simon, Volker Mailänder, Svenja Morsbach, Katharina Landfester

**Affiliations:** ^1^ Max Planck Institute for Polymer Research Ackermannweg 10 55128 Mainz Germany; ^2^ Department of Dermatology University Medical Center of the Johannes Gutenberg‐University Mainz Langenbeckstraße 1 55131 Mainz Germany

**Keywords:** clusterin, immunoglobulin G, nanocarriers, protein corona, stealth effect

## Abstract

Nanocarriers for medical applications must work reliably within organisms, independent of the individual differences in the blood proteome. Variation in the blood proteome, such as immunoglobulin levels, is a result of environmental, nutrition, and constitution conditions. This variation, however, should not influence the behavior of nanocarriers in biological media. The composition of the protein corona is investigated to understand the influence varying immunoglobulin levels in the blood plasma have on the interactions with nanocarriers. Specifically, the composition of the nanocarriers' coronas is analyzed after incubation in plasma with normal or elevated immunoglobulin G (IgG) levels, and cellular uptake is monitored in cell lines containing different immunoglobulin receptors. Here, it is reported that upon doubling the IgG concentration in plasma, the IgG fraction in the protein corona increases by a factor of 40 independent of the nanocarrier material. This results in a significant increase in uptake in cells exhibiting IgG binding receptors. Furthermore, precoating nanocarriers with clusterin successfully prevents dominant IgG‐adsorption and additionally reduces cellular internalization, after incubation with IgG‐enriched plasma. Therefore, precoating nanocarriers may be utilized as a powerful method to reduce the influence of individual variations in blood composition on the protein corona.

## 1 Introduction

Nanocarriers (NCs) are being investigated with deepening interest as potential candidates for therapeutic and diagnostic purposes. They can be loaded with drugs or reporter molecules and be selectively transported to desired cells or tissues, without exposing the cargo to the rest of the body.^1^ The NCs can be intravenously administered, where they interact with the proteins present in the blood resulting in the formation of a protein corona, which are the proteins adsorbed to the NCs' surface.^2^, ^3^, ^4^

In investigations of the protein corona, pooled blood plasma or blood serum from multiple healthy donors is usually used as biological medium to characterize the interactions of NCs and blood proteins. To complicate the situation, it has been shown that different plasma sources^5^, ^6^ or anticoagulants^7^ significantly change the corona formation process influencing the biological behavior. Consequently, pooled blood from healthy donors does not necessarily reflect individual concentration fluctuations of blood constituents, especially in patients with a disease.

The blood proteome composition of an individual can vary depending on environmental conditions, nutrition, and constitution. Specifically, immunoglobulins such as immunoglobulin G (IgG) are highly influenced by the personal state of health and an alteration of IgG levels occurs in the process of many diseases. The average immunoglobulin concentration in adults is ≈16 g L^−1^, which is equivalent to about 20% of the total blood proteome with IgG representing the major immunoglobulin class at a concentration of ≈12 g L^−1^.^8^ High IgG concentrations in the human blood are a common finding and can be caused by infections, autoimmunity, inflammation, or malignancy.^9^ IgG blood levels can be increased during immune system activation by a factor of 2 for autoimmune hepatitis,^10^ by a factor of 3 for influenza A virus infections,^11^ and even more drastically by a factor of 4 or higher for multiple myeloma.^12^

As a major protein of the human blood proteome, IgG can also be found in the protein corona of nanocarriers.^13^ In literature, IgG is described as an opsonin, meaning that when present in the corona it promotes internalization of NCs into phagocyting cells.^14^ Thus, IgG reduces the blood circulation time of nanocarriers, so that other proteins are needed in the corona to counteract this effect.

In this regard, the importance of an enrichment of clusterin (also called apolipoprotein J) in the protein corona of poly(ethylene glycol) (PEG) NCs was recently discovered. The presence of clusterin resulted in a significant reduction of unspecific cell uptake in vitro, which is termed as the “stealth effect.”^15^, ^16^, ^17^ Governing of the stealth effect by active engineering of the protein corona can be achieved via two possibilities: First, any NC can be modified by tailored surfactants forcing the adsorption of clusterin^18^ or second, the NCs can directly be precoated with clusterin by adsorption.^16^ In both cases, unspecific cellular interactions were reduced significantly after exposure to plasma. However, the influence of varying immunoglobulin levels on the effectiveness of the stealth effect has not been investigated yet. As it has been reported that IgG in the protein corona can in extreme cases even lead to pronounced aggregation of the nanocarriers,^19^ the presence of IgG should be avoided independent of the blood IgG concentration. Therefore, we have investigated the influence of elevated IgG levels on the protein adsorption on NCs that feature an enrichment of stealth proteins in “normal” plasma.

In this study, we compared the protein corona of different NCs (noncovalently PEGylated polystyrene nanoparticles (PS‐NPs) and hydroxyethyl starch nanocapsules (HES‐NCs)) in pooled “normal” plasma and in plasma exhibiting a twofold concentration of IgG. Since the IgG concentration significantly changed the composition of the protein corona, also the cellular uptake was investigated in cell lines with varying presence of IgG binding receptors: murine macrophages (RAW 264.7) and human macrophages (THP‐1) which express F_c_‐receptors, and HeLa cells which lack these receptors. The contribution of IgG receptor binding to cellular internalization was verified using F_c_ blocking experiments. Subsequently, the effect of precoating the NCs with clusterin was determined concerning corona formation as well as cellular uptake, revealing that a stealth effect could indeed be restored in the presence of high IgG concentrations in plasma.

## 2 Results and Discussion

**Figure 1:**
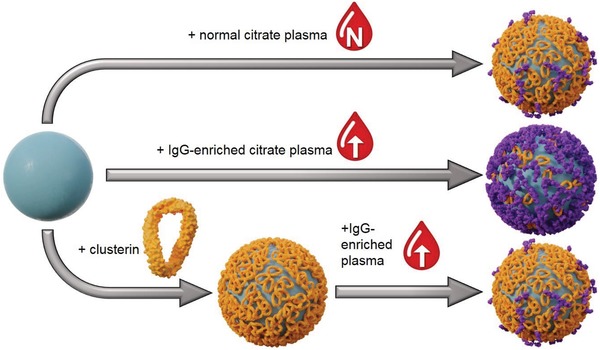
Overview of the experiment design. NCs were incubated with normal citrate plasma (top), artificially IgG‐enriched citrate plasma (middle), and artificially IgG‐enriched citrate plasma after precoating with clusterin (bottom).

In this study, interactions of different NCs with human plasma of varying IgG concentration were investigated. For this, pooled human blood plasma with averaged protein levels was obtained from healthy donors (subsequently called “normal plasma”). Furthermore, this normal plasma was modified by addition of commercially available IgG, yielding an artificially “IgG‐enriched plasma” of doubled IgG concentration, which represents a physiologically relevant concentration. For ensuring conditions close to in vivo situations, IgG extracted from human blood was used for IgG enrichment. Corona formation around NCs in the respective biological media and cellular internalization were then analyzed subsequently. Additionally, NCs were precoated with the stealth protein clusterin before incubation with IgG‐enriched plasma to evaluate the potential of negating any IgG effect. **Figure** **1** shows a schematic overview of the performed experiments.

Noncovalently PEGylated PS‐NPs and HES‐NCs were synthesized and thoroughly purified to yield minimum surfactant concentrations in the systems. Both systems are already well characterized regarding their protein corona and feature completely different surface properties, e.g., regarding their material hydrophilicity.^20^, ^21^ **Table** **1** shows transmission electron microscopy (TEM) micrographs and physicochemical characterization data for both NC systems. Besides the NCs' material, the NCs differ in morphology, size, stabilizing surfactant, and dye in order to analyze the universality of the corona formation. In Figure S1 and S2 in the Supporting Information, the autocorrelation functions of both NC systems determined from multiangle dynamic light scattering (DLS) are shown together with the extrapolated diffusion coefficients and PDIs.

**Table 1:**
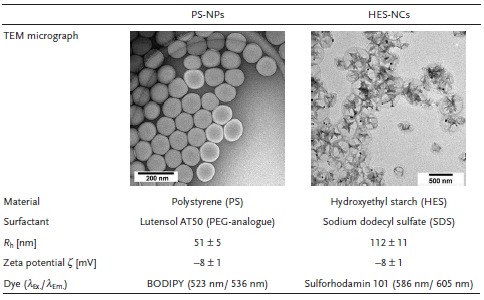
Characterization of NC systems regarding morphology and physicochemical properties

**Figure 2:**
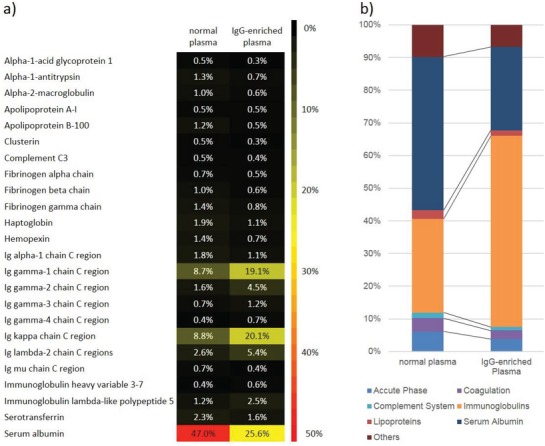
Protein composition of normal and IgG‐enriched plasma analyzed via LC‐MS. a) Heatmap indicating the most abundant proteins in the respective plasma. Only proteins, which constitute at least 0.5% of the total protein composition in one of the plasma samples with at least two unique peptides are shown. b) Bar diagram indicating the relative amount of different protein classes.

Before investigating the interaction of the NCs with the respective biological media, the used plasma samples were analyzed regarding their protein composition. Therefore, the proteome of normal plasma and IgG‐enriched plasma was determined via quantitative liquid chromatography‐mass spectrometry (LC‐MS). In **Figure** **2**a, a heatmap indicating the relative amount of the most abundant proteins is depicted. Figure 2b shows the relative amount of different protein classes (such as immunoglobulins or complement system proteins in general) in the plasma samples.

As anticipated, it can be seen that the relative protein composition of normal and IgG‐enriched plasma was different regarding individual proteins and protein classes. The relative concentration of IgG fractions was roughly doubled in the IgG‐enriched plasma (e.g., 19.1% IgG‐1 chain vs 8.7% in normal plasma). Consequently, the total fraction of immunoglobulins was increased to ≈60% in IgG‐enriched plasma (vs ≈ 30% in normal plasma). To verify, whether the IgG was still in its native form and no denaturation had occurred, the melting point was measured with nano differential scanning fluorimetry (nanoDSF), which yielded a melting point of *T*_m_ = 69.4 ± 0.1 °C (see Figure S3, Supporting Information). This is in good agreement with values reported in literature for whole IgG isotopes,^22^ while two transitions were reported for the IgG domains isolated by cleaving the connecting hinge region (*T*_m_ = 61 °C for the F_ab_ fragment and *T*_m_ = 71 °C for the F_c_ fragment).^23^ Therefore, it can be concluded, that the herein used IgG protein was in its native form.

**Figure 3:**
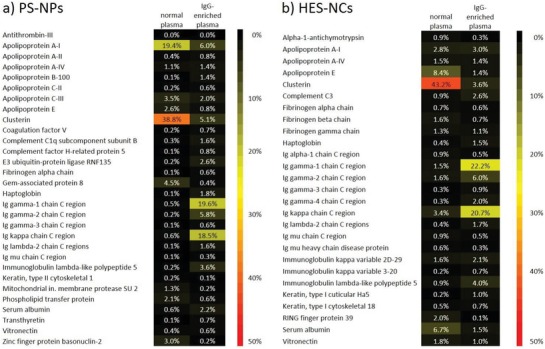
Composition of protein corona after incubation with normal and IgG‐enriched plasma analyzed via LC‐MS for a) PS‐NPs and b) HES‐NCs. Only those proteins, which constitute at least 0.5% of the protein corona on one of the nanocarriers with at least two unique peptides are shown.

Next, the protein corona formation in both plasma sources was evaluated. For this, PS‐NPs and HES‐NCs were incubated in both plasma sources (20%‐diluted plasma to ensure solubility of additional IgG, see the Experimental Section) and subsequently centrifuged and washed to remove excess free proteins. The protein composition of the protein coronas analyzed via LC‐MS is shown in **Figure** **3**a for PS‐NPs and Figure 3b for HES‐NCs. Additionally, the protein adsorption was verified by zeta potential measurements of each NC with and without protein corona obtained from the different plasmas (see Table S1, Supporting Information).

Following Figure 3, the composition of the protein corona of both NCs differs a lot for the different plasmas. For the incubation in normal plasma, clusterin was the main protein corona component, followed by apolipoprotein A‐I (PS‐NPs) and apolipoprotein E (HES‐NCs), while IgG and its fractions were not a major component of both NCs' protein corona. This agrees well with the corona composition for both systems already reported before.^19^, ^24^ However, after incubation in IgG‐enriched plasma the relative amount of IgG chains in the protein coronas was significantly increased (e.g., 19.6% IgG‐1 chain vs 0.5% for PS‐NPs). For both NC systems, the overall protein corona consists of almost 50% immunoglobulins after incubation in IgG‐enriched plasma. Clusterin and other proteins enriched before were still present, although in much lower amounts. Strikingly, the enrichment of IgG components in the protein corona was far greater than the enrichment of IgG in the plasma sample itself (enrichment by a factor of around 40 in the corona vs factor of around 2 in the plasma sample). This suggests that the interactions between NCs and plasma proteins strongly depend on the individual protein concentration (in this case IgG) in the biological medium and are not purely a result of the individual protein binding affinities. While clusterin levels in blood average around only 0.1 g L^−1^,^14^ elevated IgG levels might lead to suppression of clusterin interactions with the NCs by simply blocking its access to the surface due to the high concentration. This is in agreement with the commonly described “Vroman effect,” which defines the first step of protein adsorption as a kinetically controlled process.^25^, ^26^ According to that effect, proteins with a high abundance and high mobility may adsorb first to a nanocarrier and after longer time be replaced by proteins with a lower abundance/mobility but higher binding affinity. In our case, the initial adsorption of IgG might block the nanocarrier surface from adsorbing clusterin, which has a higher binding affinity, but very low abundance especially in IgG‐enriched plasma. Usually, the timescale of such kinetically driven changes in the protein adsorption occurs within 1 h of incubation time.^27^, ^28^, ^29^ However, for the system described here, this is apparently not the case, as the incubation time of experiments discussed in this study was 1 h as well. This might be due to the high initial concentration of IgG so that further rearrangements were outside of the experimental timeframe.

**Figure 4:**
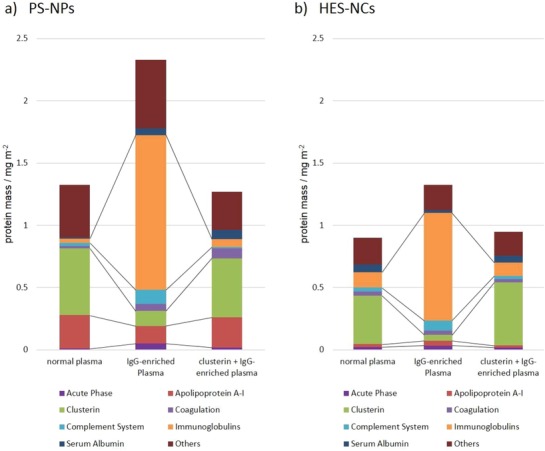
Composition of protein corona of a) PS‐NPs and b) HES‐NCs according to protein class after incubation with normal plasma, IgG‐enriched plasma or IgG‐enriched plasma after preincubation with clusterin analyzed via LC‐MS normalized for protein amounts detected by a Pierce 660 nm protein assay.

Accordingly, the dominant IgG adsorption might be prevented by previous incubation of the NCs with clusterin in order to allow a precoating of the NC surface with the desired stealth protein possessing a high binding affinity but low plasma concentration. Thus, the protein corona of both NC systems after precoating with clusterin and subsequent incubation in IgG‐enriched plasma was analyzed by LC‐MS and compared to the protein patterns shown in Figure 3. Moreover, the identified proteins were normalized by the total protein mass detected in the corona via a Pierce 660 nm quantification assay (see Figure S4, Supporting Information) to determine whether immunoglobulins replaced the apolipoproteins or were adsorbed additionally (see **Figure** **4**).

For both NCs, precoating with clusterin and subsequent incubation with IgG‐enriched plasma yielded a protein corona that was similar compared to the respective NCs, which were incubated with normal plasma, both qualitatively and quantitatively for individual protein masses and total protein adsorption. This effect of dominant IgG adsorption and its prevention by clusterin‐precoating were also observed in the analysis of the protein coronas of both NCs in the different plasmas via sodium dodecyl sulfate polyacrylamide gel electrophoresis (SDS–PAGE) (see Figures S5–S7, Supporting Information). Without the precoating of NCs with clusterin, the total mass of proteins adsorbed on the respective NCs increased significantly after incubation with IgG‐enriched plasma (see Figure S4, Supporting Information). This resulted not only in the previously discussed relative enrichment of IgG fractions in the corona but also a higher absolute mass of immunoglobulins and a lower absolute mass of apolipoproteins (clusterin and apo A‐I) was found on the NCs. Therefore, it can be concluded that a replacement of these apolipoproteins by IgG took place in IgG‐enriched plasma, which could be prevented by precoating with clusterin. The LC‐MS (Figure 4) and SDS–PAGE (Figures S5–S7, Supporting Information) results show near identical amounts of clusterin in the corona of both NPs incubated with normal plasma and IgG‐enriched plasma after clusterin pretreatment. This raises the possibility that the corona is saturable with clusterin, and saturation is reached by a dose less than the externally added amount. Interestingly, the corona composition after clusterin precoating is very similar to the corona in normal plasma, which could be explained by the Vroman effect as described above: Clusterin has one of the highest binding affinities but a very low abundance. If it is allowed to interact with the nanocarriers first, it is possible that it actually reaches the surface. Afterwards, the equilibrium can form also containing all other proteins because it is not kinetically hindered so much concerning the high IgG concentration.

In addition to the two NC systems described here, the effect of IgG‐enrichment in the protein corona postincubation with IgG‐enriched plasma was also found for several other different nanomaterials (Table S2 and Figures S8–S12, Supporting Information), verifying that the effect is not specific to certain materials. In the following, however, we concentrated on the investigation of the already characterized PS‐NPs and HES‐NCs.

**Figure 5:**
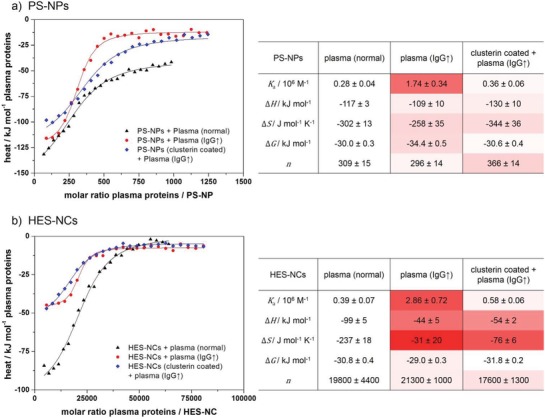
Adsorption isotherms and parameters of plasma proteins titrated to a) PS‐NPs and b) HES‐NCs obtained from ITC experiments. The average molar concentration of plasma proteins was calculated by dividing the mass concentration of plasma proteins by the mean molar mass of all plasma proteins according to their relative contribution determined via LC‐MS (see details in the Supporting Information). Isotherms were fitted according to an independent binding model (solid lines). Color code indicates relative deviation (white = no deviation, red = strong deviation) of adsorption parameters from the parameters obtained from titrations with normal plasma (second column of each table).

Since the IgG level of the plasma had a large impact on the formation of the hard protein corona as observed via LC‐MS, further information about the total interaction (including the soft corona) between NCs and the different plasmas were of high interest. Therefore, calorimetric measurements of both NCs were performed via isothermal titration calorimetry (ITC) to obtain information about the overall influence of the IgG on interaction thermodynamics. In each experiment, normal or IgG‐enriched plasma was titrated into a suspension of the respective NCs (with or without precoating with clusterin). Additionally, the heat of dilution of plasma (titration of plasma into buffer) was determined and subtracted from the initial measurement. The obtained corrected injection heats were fitted with an independent binding model. For this, the total molar concentration of plasma proteins was calculated based on the composition determined by LC‐MS (see the Supporting Information for further details). **Figure** **5** shows the resulting adsorption isotherms and parameters of these ITC experiments.

Certainly, it has to be pointed out that all obtained adsorption parameters present an average over all involved proteins and processes so that the absolute values cannot be interpreted reliably. However, the relative values obtained for the different adsorption scenarios (1) normal plasma, 2) IgG‐enriched plasma, and 3) clusterin preincubation + IgG‐enriched plasma) can be compared to each other. According to Figure 5, it is clear that the adsorption parameters of plasma proteins in normal and IgG‐enriched plasma significantly deviate from each other for both systems. Significantly higher average association constants *K*_a_ were obtained for the titration of uncoated NCs with IgG‐enriched plasma (indicated by the color coding in the tables), meaning that overall the affinity to bind plasma proteins is higher. This correlates with the elevated level of IgG in the NCs' corona as demonstrated via LC‐MS and the fact that the overall amount/mass of adsorbed protein is higher in IgG‐enriched plasma (compare Figure 4). When the NCs were precoated with clusterin and subsequently titrated with IgG‐enriched plasma, the observed average binding affinities were decreased again and similar to the parameters in normal plasma. From the other parameters such as enthalpy and entropy, in principle information about the driving force of the interactions can be concluded. Δ*H* and Δ*S* change similar to the binding affinity when changing the plasma source. This is expected because as the protein corona composition changes also the involved interaction mechanisms might differ (electrostatic interactions, hydrogen bonding, etc.). When introducing the clusterin precoating, Δ*H* and Δ*S* again change in the titration with IgG‐enriched plasma, but do not completely go back to the values obtained in normal plasma. This is especially the case for the HES‐NCs and probably a result of the fact that HES is a more hydrophilic material compared to PS. Therefore, a larger contribution of more loosely bound corona proteins is expected, which would only be visible in the ITC experiments as no washing takes place.^24^ While loosely bound proteins are mainly removed from the NCs in the washing steps during corona preparation (as prepared for MS experiments), adsorption of all proteins are measured in ITC including proteins of lower affinity. Accordingly, we conclude from the ITC experiments that increasing the IgG level in plasma indeed also changes the thermodynamic parameters and, thus, the mechanism of interaction of the proteins overall.

**Figure 6:**
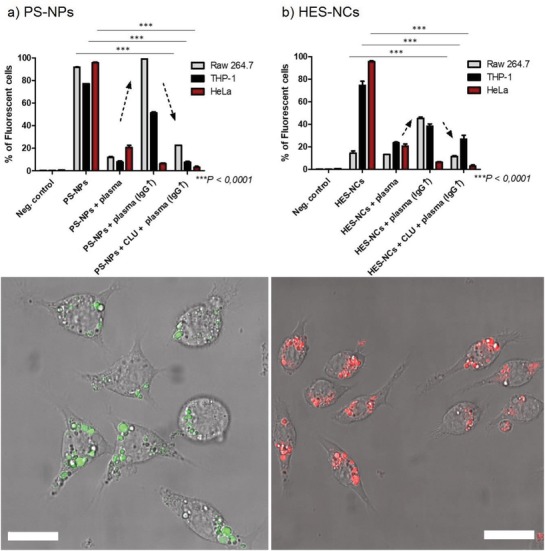
Top: Cellular uptake of a) PS‐NPs and b) HES‐NCs before and after incubation with normal plasma, IgG‐enriched plasma, and clusterin followed by IgG‐enriched plasma. RAW 264.7 (murine macrophages), THP‐1 (human macrophages), and HeLa cells (human cancer cells) were used as cell lines. For the negative control, only cells in medium were measured without addition of sample. Values are mean values with standard deviation of three biological replicates. The analysis of variance (ANOVA) two‐way test was used for statistical analysis yielding ***p < 0.0001 corresponding to the individual types of NCs. Arrows indicate the increased cell uptake after incubation in IgG‐enriched plasma and decreased uptake after preincubation with clusterin. Bottom: Pictures of a) PS‐NPs (pseudocolored in green) and b) HES‐NCs (pseudocolored in red) without protein corona in RAW 264.7 cells. Pictures were chosen to distinguish cellular uptake from cell membrane decoration. The scale bar corresponds to a length of 20 µm.

As we observed a significant effect of elevated IgG levels in plasma on the protein corona formation, the influence of this altered protein corona on cellular uptake of different cell lines was investigated subsequently. The aim of this was to figure out if NCs with increased IgG concentration in their corona would exhibit an elevated endocytosis mediated by F_c_ receptors, which specifically bind the F_c_ region of antibodies. For this, both NCs with the different protein coronas discussed before were incubated with macrophages that express F_c_ receptors (human macrophage cell line: THP‐1 or mouse macrophage cell line: RAW 264.7) and HeLa cells (human cancer cell line), which lack F_c_ receptors (see **Figure** **6**).^30^ No additional proteins were added to the cell culture medium. For reference, NCs without protein corona were incubated with each cell line. Cellular uptake was verified via confocal laser scanning microscopy (cLSM, see Figure 6 and Figures S13–S18, Supporting Information). Cell viability was verified with Zombie Aqua viability kits (see Figure S19, Supporting Information).

According to the flow cytometry results, pristine NCs (without protein corona) were readily taken up by the different cells, while NCs incubated with normal plasma showed significantly decreased uptake due to their protein corona with the exception of HES‐NCs in RAW 264.7 cells. In contrast, after incubation in IgG‐enriched plasma both NCs showed a significantly increased uptake in RAW 264.7 and THP‐1 cells while uptake in HeLa cells was slightly decreased. Precoating with clusterin before incubation in IgG‐enriched plasma again reduced the cellular uptake for both systems to a similar level as with the “normal” protein corona. We suggest that the increased uptake of uncoated NCs incubated with IgG‐enriched plasma was due to the increased IgG concentration in the corona, as RAW 264.7 and THP‐1 cells express F_c_‐receptors, which HeLa cells lack completely.

**Figure 7:**
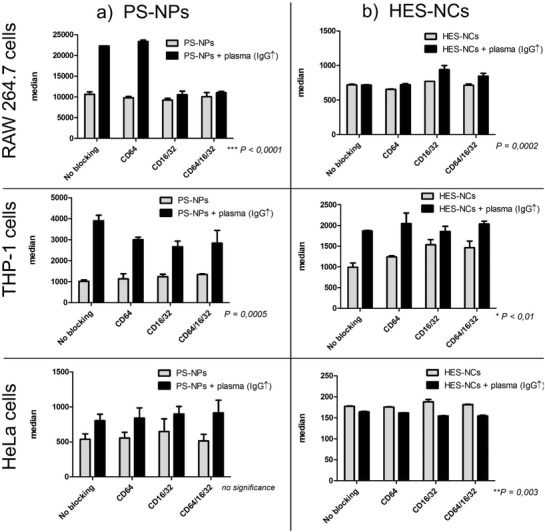
F_c_ blocking experiments of a) PS‐NPs and b) HES‐NCs before and after incubation with IgG‐enriched plasma. RAW 264,7 (murine macrophages), THP‐1 (human macrophages), and HeLa cells (human cancer cells) were used as cell lines. Values are mean values with standard deviation of three biological replicates. The ANOVA two‐way test was used as statistical analysis. *P*‐values describe the interaction between NCs (without protein corona or with corona from IgG‐enriched plasma) and blocked/unblocked receptors. CD16/CD32 (binding aggregated IgG with low affinity for the ligand) and CD64 (binding monomeric IgG with high affinity for the ligand) receptors were either blocked individually or all three at the same time.

In order to further investigate the uptake mechanism of the NCs regarding IgG specificity, F_c_ blocking experiments were performed in which the different F_c_ receptors were blocked by adding purified anti‐CD64, anti‐CD16, and/or anti‐CD32 before incubation with nanocarriers. For determining the exact uptake pathway, the receptors CD16/CD32 (binding aggregated IgG with low affinity for the ligand^30^) and CD64 (the only receptor that can bind monomeric IgG and has high affinity for the ligand^31^) were blocked individually and additionally all three at the same time. The results of these blocking experiments are depicted in **Figure** **7** showing the median of fluorescence intensity (% fluorescent cells see Figure S20, Supporting Information).

As it can be seen, the uptake of NCs without protein corona did not change significantly when any F_c_‐receptors were blocked. However, for PS‐NPs incubated with IgG‐enriched plasma the uptake was reduced to the same level as for PS‐NPs without protein corona in RAW 264.7 cells when CD16/CD32 were blocked, suggesting the binding to these receptors to be majorly responsible for the initially increased uptake.^14^ This is in good agreement with differential scanning flourimetry (nanoDSF) experiments showing that IgG exists in denatured configuration on the surface of PS‐NPs (see Figure S21, Supporting Information). Only blocking CD64, however, did not affect the uptake. In THP‐1 cells, the uptake of PS‐NPs in IgG‐enriched plasma was decreased significantly when CD16/CD32 and/or CD64 were blocked. The uptake was not decreased to the level of bare NCs though, highlighting that human macrophages are more complex than mouse macrophages. Blocking all three F_c_‐receptors in THP‐1 cells still showed an uptake higher than the negative control so that the uptake mechanisms for these NCs are unclear. Experiments with the macrophage cell lines for HES‐NCs showed no significant decrease of uptake which is most likely linked to the generally low absolute uptake of these NCs as observed in the median fluorescence values (≈700 for HES‐NCs vs ≈10 000 for PS‐NPs). Uptake in HeLa cells was not affected by blocking for any of the NCs, which is in accordance with the fact that these cells lack respective receptors.

Summing up the in vitro experiments, cellular uptake strongly depended on the composition of the protein corona while the uptake mechanisms differed based on the NCs' material and cell type. While the uptake of PS‐NPs was mediated by aggregated IgG via the CD16/CD32 receptors, the uptake of HES‐NCs seemingly was not connected to this pathway. This could potentially be related to the accessibility of the antibodies' F_c_ regions on the surface of the different NCs. It could be possible that IgG bound with different orientation on the respective NCs resulting in the recognition of its F_c_ region on PS‐NPs, which is not an apparent pathway of internalization for HES‐NCs. Also, it could be that other proteins adsorbed on the HES‐NCs shielded the F_c_ parts of the IgGs in a different way than for the PS nanocarriers. The IgG enrichment and effect in the protein corona was lower for the HES‐NCs than for the PS‐NPs and can be prevented by precoating with clusterin, negating the hard to predict outcome for cellular uptake.

## 3 Conclusion

In this study, we have shown the influence of varying immunoglobulin blood plasma levels on the formation of the protein corona. The protein corona composition and resulting cellular response for polymeric nanocarriers exposed to blood plasma with normal and artificially elevated IgG levels was characterized regarding the protein corona composition and subsequent effect on cellular internalization. Strikingly, upon doubling the IgG concentration in plasma, the fraction of IgG in the respective coronas was elevated by a factor of 40, while the adsorption of stealth proteins such as clusterin was promoted for nanocarriers in normal plasma. This IgG enrichment in the protein corona led to significantly increased uptake in human and murine macrophages via F_c_‐receptor mediated endocytosis, as supported by F_c_ blocking experiments. Precoating of the nanocarriers with clusterin before protein corona formation successfully prevented dominant IgG‐adsorption and additionally reduced cellular internalization. Following this, precoating with clusterin or potentially other stealth proteins with high binding affinities can be regarded as a powerful method to reduce the influence of individual blood composition variations (e.g., as an outcome of different health states) on the biological identity of nanocarriers. Ultimately, we suggest that nanocarriers can be precisely engineered using “body‐own” materials such as the blood proteins to achieve a universal performance. In our opinion, this methodology could pave the way to gain control over the behavior of nanocarriers in biological media and, thus, allow for more successful translation of nanocarriers into the clinics.

## 4 Experimental Section

*Materials*: IgG from human serum was purchased from Sigma‐Aldrich (St. Louis, USA) and used without further purification. Clusterin from human plasma was purchased from BioVendor (Brno, Czech Republic) and used without further purification. Blood was taken at the University Medical Centre of the Johannes Gutenberg University Mainz from ten healthy donors after obtaining informed consent. All experiments containing human blood plasma from these donors were approved by the ethics committee of the Landesärztekammer Rheinland‐Pfalz, Mainz No. 837.439.12 (8540‐F) and thus performed in compliance with all relevant laws and guidelines. The blood was centrifuged and the plasma supernatant was pooled, aliquoted, and stored at ‐80 °C. The thawed plasma was centrifuged at 20 000 g for 1 h at room temperature in order to remove residual protein precipitates before usage. For preparation of the protein corona, 200 µL of freshly thawed and centrifuged plasma were diluted with 800 µL of phosphate buffered saline (PBS), resulting in 20% diluted plasma. For experiments with IgG‐enriched plasma, 6 mg of IgG was dissolved in 1 mL of diluted plasma.

The herein used PS‐NPs were synthesized using a miniemulsion polymerization method with Lutensol AT50 (a poly(ethylene oxide)‐hexadecyl ether with an ethyleneoxide length of about 50 units) as surfactant and the used HES‐NCs were synthesized using a miniemulsion polymerization method with sodium dodecyl sulfate (SDS) as surfactant as previously published.^32^ The used nanoparticles were filtered through Millex‐SV 5 µm filters (Merck Millipore, Billerica, USA) before use in order to remove aggregates or potential impurities like dust. After synthesis, the particles were centrifuged and resuspended (five times in the case of PS‐NPs and two times for the HES‐NCs) and dialyzed for 4 h to remove excess surfactant from the particles' surface. Minimal amounts of surfactant remained in the nanoparticle dispersion for preventing agglomeration of the nanomaterials.

*Corona Preparation*: For each sample, an aqueous nanoparticle suspension (0.05 m² of surface area in a total volume of 300 µL) was mixed in an Eppendorf‐tube with 1 mL of the respective diluted plasma. For precoating, samples were incubated with 66 µL of an aqueous clusterin solution (20 mg L^−1^ in PBS) for 10 min at 37 °C before addition of IgG‐enriched plasma. After 1 h of mild shaking at 37 °C, the sample was centrifuged for 1 h at 20 000 g and 4 °C. The supernatant was discarded and the pellet resuspended in 1 mL of PBS. The suspension was again centrifuged for 1 h at 20 000 g and 4 °C. These washing steps were repeated for a total of three times. Before the last washing step, the suspension was transferred into a new Eppendorf‐tube. After the last washing step, the pellet was resuspended in 1 mL of ultrapure water.

*LC‐MS*: LC‐MS analysis of protein samples was carried out as described in a previous publication.^33^ A nanoACQUITY ultra high‐performance liquid chromatography (UPLC) system coupled with a Synapt G2‐Si mass spectrometer (Waters Corporation) was used. A nanoACQUITY system equipped with a C_18_ analytical reversed‐phase column (1.7 µm, 75 µm x 150 mm, Waters Corporation) and a C_18_ nanoACQUITY Trap Column (5 µm, 180 µm x 20 mm, Waters Corporation) were used to separate the tryptic‐digested peptides originating from 25 µg total protein. Peptide separation was done with a mobile phase A consisting of 0.1% (v/v) formic acid in water and a mobile phase B consisting of acetonitrile with 0.1% (v/v) formic acid at a flow rate of 0.3 µL min^−1^, using a gradient of 2–40% mobile phase B for 70 min. 150 fmol µL^−1^ Glu‐Fibrinopeptide was infused at a flow rate of 0.5 µL min^−1^ as a reference compound. Data‐independent acquisition mean squared error (MSE) experiments were done on the Synapt G2‐Si in resolution mode. Electrospray Ionization was carried out in positive ion mode using a NanoLockSpray source. Data acquisition was performed in a range of m/z 50–2000 Da with one second scan time and ramped trap collision energy from 20 to 40 V with a total acquisition time of 90 min. All samples were analyzed in two technical replicates. Data were acquired and processed using MassLynx 4.1 and Progenesis QI for proteomics v2.0 software. Data were lock mass corrected after acquisition. As noise reduction thresholds for low energy, high energy, and peptide intensity, 120, 25, and 750 counts were used. The protein false discovery rate was set at 4% in database searches. The generated peptide masses were compared to a reviewed human protein sequence database downloaded from Uniprot. As search criteria, one missed cleavage, maximum protein mass 600 kDa, fixed carbamidomethyl modification for cysteine, and variable oxidation for methionine were applied. A peptide required at least two assigned fragments and a protein required at least two assigned peptides and five assigned fragments in order to be identified. Quantitative data were generated based on the TOP3/Hi3 approach, providing the amount of each protein in fmol. The protein corona was prepared as described above.

*Zeta Potential*: Zeta potential measurements were performed using a Nano Z Zetasizer (Malvern Instruments GmbH, Herrenberg, Germany). 20 µL of each sample as obtained after the procedure described for the corona preparation were diluted with 1 mL of a 1 × 10^−3^ m KCl solution and measured instantly at 25 °C after two minutes of equilibration. Each measurement was repeated in triplicate and mean values as well as standard deviations were calculated.

*DLS*: DLS measurements were performed using an instrument from ALV (Langen, Germany) consisting of an electronically controlled goniometer and an ALV‐5000 multiple τ full‐digital correlator with 320 channels with a measurement range between 10^−7^ s and 10^3^ s. A helium‐neon laser (Type 1145 P) from JDS Uniphase (Milpitas, USA) of 632.8 nm wavelength and 25 mV output power was used as source of light. Before measurements, samples were filtered into quartz cuvettes for light scattering from Hellma (Müllheim, Germany) with an inner radius of 9 mm. Millex‐SV filters (Merck Millipore, Billerica, USA) with 5 µm pore size were used. Prior to use, the quartz cuvettes were cleaned with acetone using a Thurmond apparatus.^34^

*TEM*: TEM micrographs were taken on an FEI Tecnai F20 transmission electron microscope operated at 200 kV. Micrographs were taken using a 2k charge‐coupled device camera from Gatan (Type: Ultrascan 1000).

*nanoDSF*: NanoDSF measurements of IgG solutions with or without presence of PS‐NPs were performed using a NanoDSF Prometheus NT.48 device with standard capillaries (NanoTemper Technologies, München, Germany). The IgG concentration in each sample containing IgG was 1.0 g L^−1^. Analysis and online monitoring of the DSF measurement was performed using the PR.Controll Data Analysis Software (v1.12.3) from NanoTemper Technologies. Fluorescence of each sample was analyzed at a wavelength of 350 nm and 330 nm. The temperature was increased from 20.0 °C to 95.0 °C at a rate of 0.5 °C min^–1^.

*ITC*: ITC measurements were performed using a NanoITC Low Volume (TA Instruments, Eschborn, Germany) with an effective cell volume of 170 µL. During each experiment 50 µL of the respective plasma were titrated into 300 µL of an aqueous suspension of PS‐NPs (*c* = 5.3 × 10^−5^ mm) or 300 µL of an aqueous suspension of HES‐NCs (*c* = 8.2 × 10^−7^ mm) respectively. The concentration of NCs was determined from the solid content by assuming spherical particles/capsules and applying the respective radius determined via DLS, the material density and in the case of HES‐NCs the shell thickness of the capsules. Additionally, the same amount of plasma solution was titrated into 300 µL of ultrapure water for determining the dilution heat for reference. The number of injections was set to 25 for each measurement (25 × 2 µL) with a spacing of 250 s between every injection. Each measurement was carried out at 15 °C. The integrated heats of dilution were subtracted from the integrated heats of every adsorption measurement. The normalized heats were fitted according to an independent binding model (see Equation (S1), Supporting Information) to obtain the association constant (*K*_a_), the reaction enthalpy (Δ*H*), the entropy (Δ*S*), the Gibbs free energy (Δ*G*), and the reaction stoichiometry (*n*). Each measurement was carried out in triplicate and the mean value as well as standard deviation for each parameter were calculated. Data evaluation of the ITC measurements was performed using the Nano Analyze Data Analysis Software (Software version 2.5.0) from TA Instruments.

*Cell Culture*: The murine macrophage cell line RAW 264.7, the human HeLa, and THP‐1 cell lines were maintained in Roswell Park Memorial Institute (RPMI)‐1640 medium supplemented with 10% fetal calf serum and 100 U mL^−1^ penicillin (all from Gibco, Germany) at 37 °C with 5% CO_2_ in an incubator.

*THP‐1 Macrophage Differentiation*: The human monocyte cell line THP‐1 was differentiated into macrophages prior the experiments with the nanocarriers for 5 d. On day 0 the cells were stimulated with 100 ng mL^−1^ of phorbol 12‐myristate 13‐acetate (PMA) (Sigma‐Aldrich, Germany) and seeded at a density of 150 000 cells per well in 24‐well plates. After 2 d, the medium was changed to fresh RPMI without PMA and the cells rested for the following 3 d before the experiment.

*Cell Blocking Experiments with Antibodies*: For the cell blocking experiments, purified anti‐CD64, CD16, and/or CD32 (BioLegend, USA) were added to the cells at 5 µg mL^−1^ in fresh serum‐free medium for 30 min at 37 °C before the respective samples. After the incubation, the nanoparticles were added to the wells and the cells incubated according to the time points described below. No wash step was performed after the incubation with the antibodies.

*Cell Uptake Experiments and Flow Cytometry Measurements*: For the cell uptake experiments, cells were seeded at a density of 150 000 cells per well in 24‐well plates. The cells were incubated in fresh serum‐free medium with the nanocarrier dispersions added at a concentration of 75 µg mL^−1^ to the cells for 2 h (RAW 264.7 and THP‐1) or 16 h (HeLa cells). For flow cytometry experiments, adherent cells were washed with PBS and detached from the culture vessel with 2.5% trypsin (Gibco, Germany) and measurements were performed on the Attune Nxt cytometer (Invitrogen, Germany) with a 488 nm laser for excitation of boron‐dipyrromethene dye (BODIPY) and a 561 nm laser for excitation of Sulforhodamine. The viability of the cells was measured by staining with the viability dye Zombie Aqua (BioLegend, USA) according to the manufacturer's instruction, prior the flow cytometry measurements. The 405 nm laser was used for the excitation of the Zombia Aqua dye.

*SDS–PAGE*: After the last washing step of the corona preparation (see above), the pellet was suspended in 100 µL of a 62.5 × 10^−3^ m Tris*HCl solution containing 2% of SDS. The suspension was incubated at 95 °C for 5 min and was centrifuged for 1 h at 20 000 g and 4 °C. The protein concentration of each sample was determined using a Pierce 660 nm Assay Kit by ThermoFisher (Waltham, USA) with bovine serum albumin as standard reagent as described by the manufacturer. 26 µL of the supernatant containing 1 µg of the desorbed proteins according to the performed Pierce assay were mixed with 4 µL of reducing agent and 10 µL of sample buffer. Freshly thawed plasma (normal or IgG‐enriched respectively) were used as reference samples. After 1 h at 100 V, the electrophoresis was stopped. Staining was performed using a SilverQuest staining Kit (Invitrogen; Carlsbad, USA) as described by the manufacturer.

## Conflict of Interest

The authors declare no conflict of interest.

## Supporting information

Supplementary
