# Supplementary material for: Prevention of Dominant IgG Adsorption on Nanocarriers in IgG‐Enriched Blood Plasma by Clusterin Precoating
Source: Adv Sci (Weinh). 2019 Apr 4;6(10):1802199. doi: 10.1002/advs.201802199 (PMC6523372; doi:10.1002/advs.201802199)
Supplement: Supplementary file 1 — Supplementary [file ADVS-6-1802199-s001.pdf]

Copyright WILEY-VCH Verlag GmbH & Co. KGaA, 69469 Weinheim, Germany, 2019.

ADVANCED SCIENCE Open Access

Supporting Information

for *Adv. Sci.,* DOI: 10.1002/advs.201802199

Prevention of Dominant IgG Adsorption on Nanocarriers in IgG-Enriched Blood Plasma by Clusterin Precoating

Domenik Prozeller, Jorge Pereira, Johanna Simon, Volker Mailänder, Svenja Morsbach, and Katharina Landfester*

Supporting Information

Prevention of dominant IgG adsorption on nanocarriers in IgG-enriched blood plasma by clusterin precoating

Domenik Prozeller,[1] Jorge Pereira[1], Johanna Simon[1,2], Volker Mailänder[2,1], Svenja Morsbach[1,*] and Katharina Landfester[1]

[1] Max Planck Institute for Polymer Research, Ackermannweg 10, 55128 Mainz, Germany

[2] Department of Dermatology, University Medical Center of the Johannes Gutenberg-University Mainz, Langenbeckstraße 1, 55131 Mainz, Germany

Corresponding author: morsbachs@mpip-mainz.mpg.de

Contents:

- Concentration calculations and ITC data analysis
- Dynamic light scattering data
- Zeta potential measurements
- Differential scanning fluorimetry (nanoDSF) data
- Pierce protein assay data
- SDS-PAGE experiments of protein corona formation for PS-NPs, HES-NCs and additional systems
- Additional cLSM pictures of cell uptake experiments
- Cell viability tests
- Additional blocking experiment figures

Calculation and data analysis

Calculation for molar concentration of plasma proteins:

For calculating the molar concentration of proteins in plasma, the mean molar mass of the most common proteins (> 0.3% via LC-MS) in the used citrate plasma was calculated by multiplying the relative amount of protein via LC-MS with the molar mass of the respective protein. Following this, a mean molar mass of 63.0 kDa was calculated.

The protein mass concentration of the plasma was determined using a Pierce 660 nm Assay Kit by ThermoFisher (Waltham, USA) with bovine serum albumin (BSA) as standard reagent as described by the manufacturer. The determined protein concentration of plasma was 48.6 g L^{-1}.

Dividing the protein mass concentration of plasma (48.6 g L^{-1}) by the mean molar mass of protein (63.0 kDa) yields the average molar concentration for plasma proteins of 0.771 mM for undiluted plasma or 0.154 mM for 20% diluted plasma as used for corona preparation.

IgG-enriched plasma was prepared by adding 6 mg of IgG (M = 150 kDa) to 1 mL of 20%-diluted plasma. This yields a concentration of IgG of 0.040 mM and results in a total molar concentration of IgG-enriched plasma of 0.194 mM.

Data evaluation for ITC experiments

Analysis of adsorption isotherms obtained by ITC experiments was performed using a fit according to an independent binding model (see equation S1).[1, 2] For this model it is assumed that a ligand L binds one site of a macromolecule M independently and without any cooperativity effects.

$$\Delta q = \left(\frac{(n[M]K_a + [L]K_a + 1) - \sqrt{(n[M]K_a + [L]K_a + 1)^2 - 4NK_a{}^2[M][L]}}{2K_a} \right) - [ML]_{n-1} \Delta \mathrm{H} \Delta V_{cell} \tag{S1}$$

Following equation S1, the stoichiometry n, the association constant K_a and the binding enthalpy ΔH are determined. [M] is given as the concentration of the macromolecule, [L] as the concentration of the ligand, [ML] as the concentration of the formed complex and ΔV_{cell} as the change of the total cell volume during the titration. All values given in the main manuscript are an average of triplicates with the standard deviation of all three experiments. The Gibbs free energy is calculated following the reaction isotherm equation (S2), whereas the change in entropy ΔS is calculated by combining equation S2 with the Gibbs-Helmholtz equation (S3) and solving for ΔS (S4).

$$\Delta G = -RT \cdot \ln K_a \tag{S2}$$

$$\Delta G = \Delta H - T \cdot \Delta S \tag{S3}$$

$$\Delta S = R \cdot \ln K_a + \frac{\Delta H}{T} \tag{S4}$$

ΔG is the Gibbs free energy, R is the universal gas constant and T the temperature, so the entropy change ΔS can be calculated for known K_a and ΔH.

Supplementary Figures and Tables

a) b)

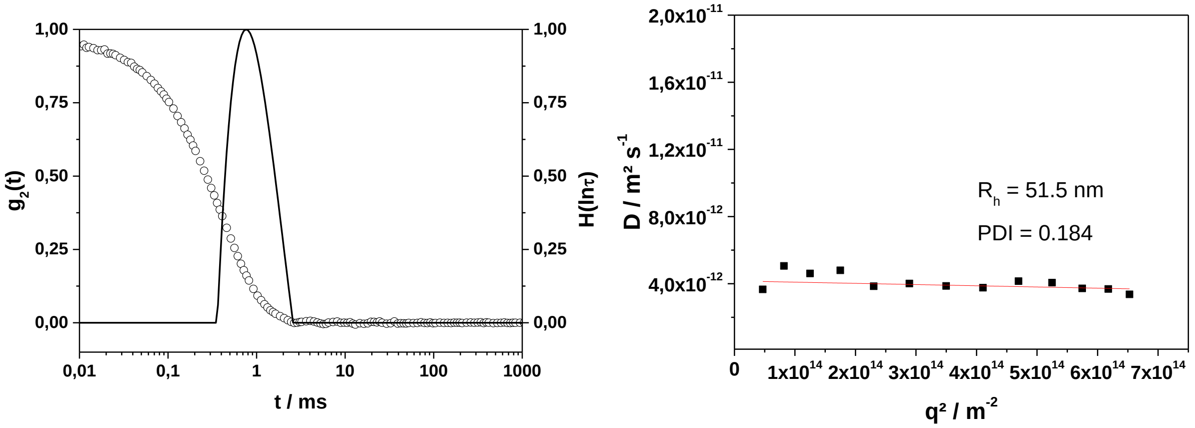

Figure S1 a) Intensity autocorrelation function $g_2(t)$ (circles ○) and relaxation time distribution H(ln τ) resulting for PS-NPs at a scattering angle of 90° (q = 0.014 nm^{-1}) from a CONTIN fit[3] (straight lines –) and b) diffusion coefficient *D* against scattering vector squared q^2. From the angular dependent, extrapolated diffusion coefficient *D*, a value of 51.5 nm was obtained for the hydrodynamic radius via the Stokes-Einstein-equation. The polydispersity index (PDI) was obtained using the cumulant fit model and was determined to be 0.184 at a scattering angle of 90°.

a) b)

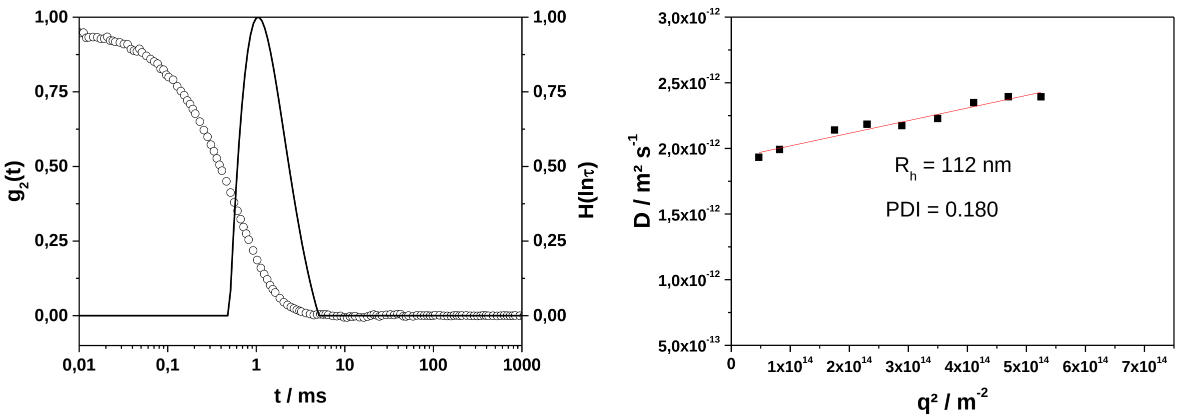

Figure S2: a) Intensity autocorrelation function $g_2(t)$ (circles ○) and relaxation time distribution H(ln τ) resulting for HES-NCs at a scattering angle of 90° (q = 0.014 nm^{-1}) from a CONTIN fit[3] (straight lines –) and b) diffusion coefficient *D* against scattering vector squared q^2. From the angular dependent, extrapolated diffusion coefficient *D*, a value of 112 nm was obtained for the hydrodynamic radius via the Stokes-Einstein-equation. The polydispersity index (PDI) was obtained using the cumulant fit model and was determined to be 0.180 at a scattering angle of 90°.

Table S1: Zeta potential of nanocarriers before and after incubation with different citrate plasmas measured in a 1 mM KCl solution.

	zeta potential of PS-NPs / mV	zeta potential of HES-NCs / mV
NCs alone	- 8 ± 1	- 8 ± 1
NCs + plasma (normal)	- 25 ± 1	- 19 ± 1
NCs + plasma (IgG-enriched)	- 15 ± 1	- 13 ± 1
NCs + clusterin + plasma (IgG-enriched)	- 27 ± 2	- 23 ± 2

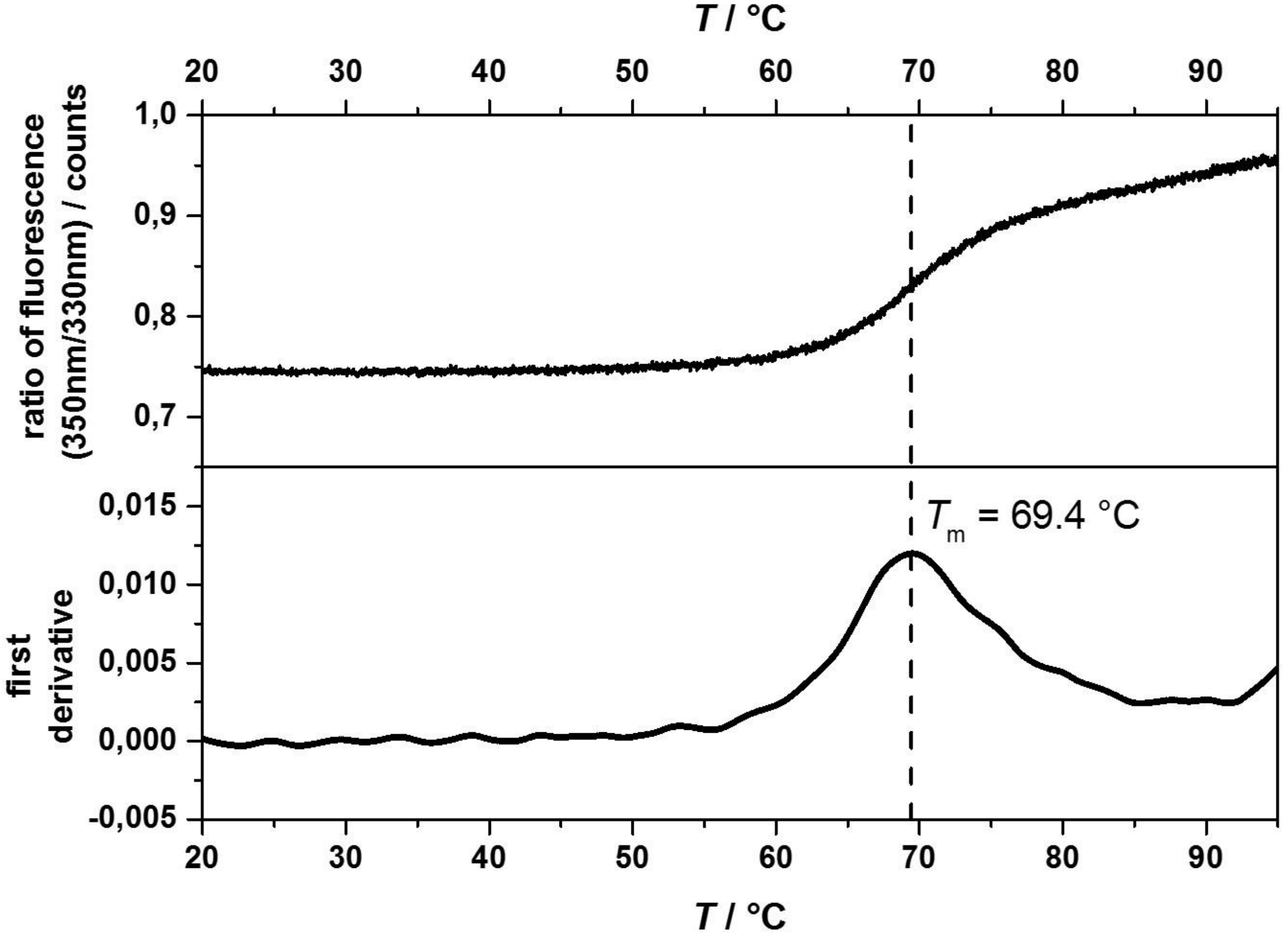

Figure S3: Differential scanning fluorimetry (DSF) of immunoglobulin G (IgG) showing the protein unfolding (heating): 350 nm / 330 nm ratio of fluorescence of IgG together with the first derivative. The melting point of native IgG (black line —) was observed at T_m = 69.4 °C.

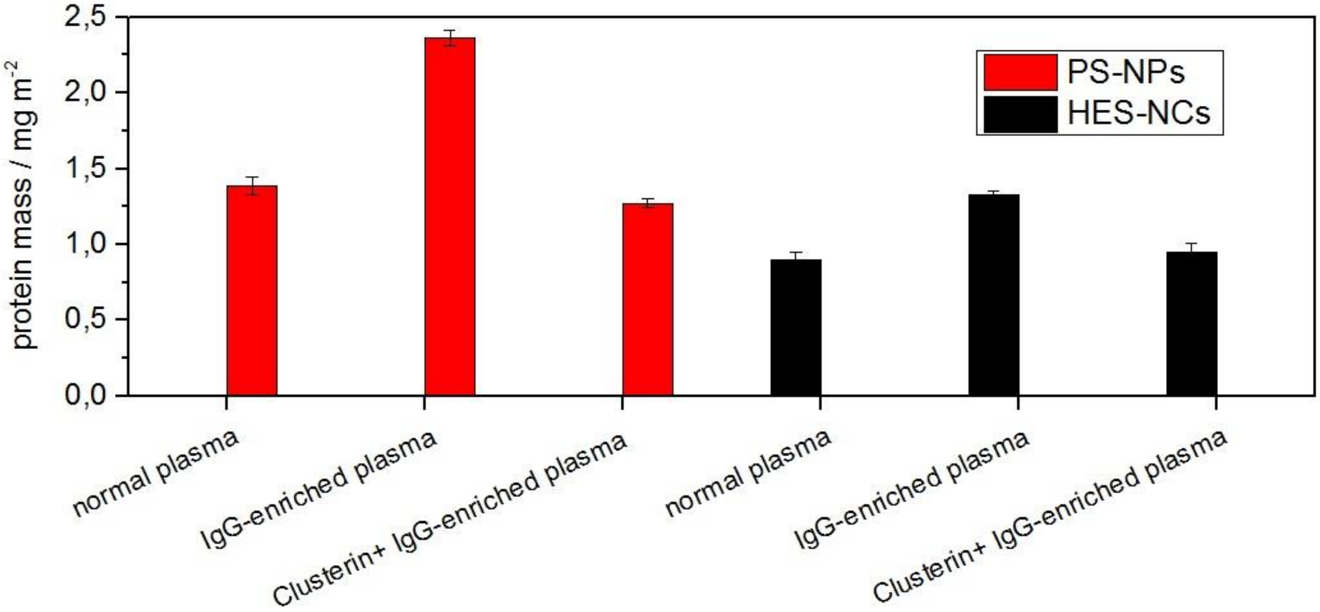

Figure S4: Protein mass on the surface of a) PS-NPs and b) HES-NCs after incubation with normal plasma, IgG-enriched plasma or IgG-enriched plasma after pre-incubation with clusterin detected by a Pierce 660 nm protein assay.

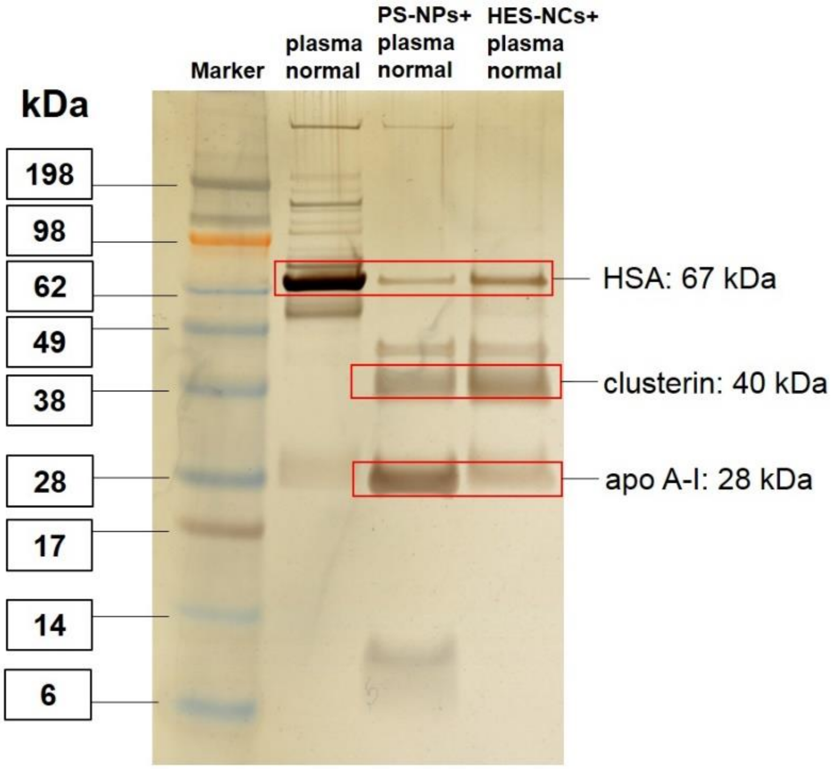

Figure S5: SDS-PAGE gel of the protein coronas of PS-NPs and HES-NCs incubated with normal plasma. Pure plasma is shown as a reference. For staining, a silver staining kit was used according to manufacturer’s instruction.

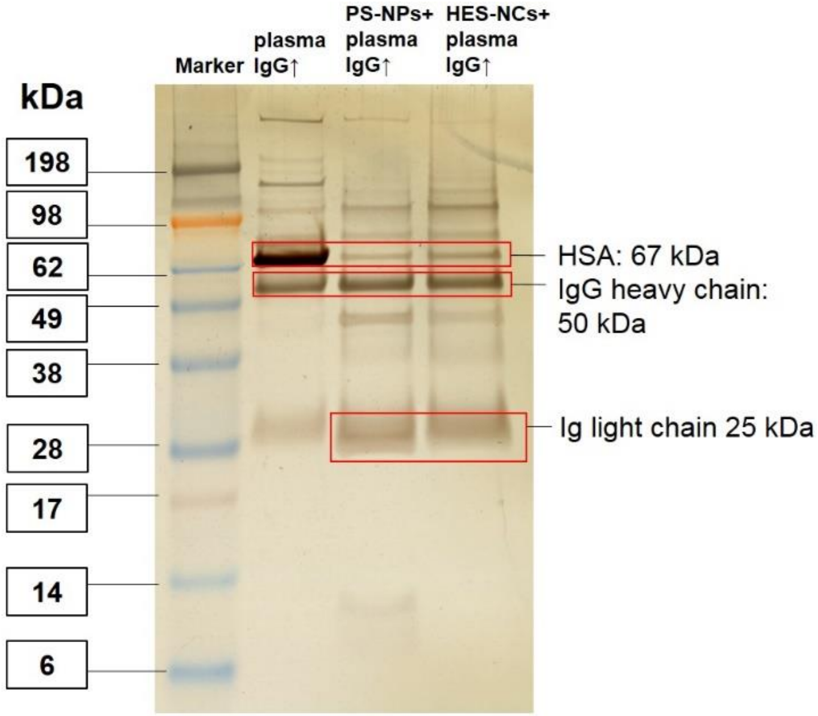

Figure S6: SDS-PAGE gel of the protein coronas of PS-NPs and HES-NCs incubated with IgG-enriched plasma. Pure IgG-enriched plasma is shown as a reference. For staining, a silver staining kit was used according to manufacturer's instruction.

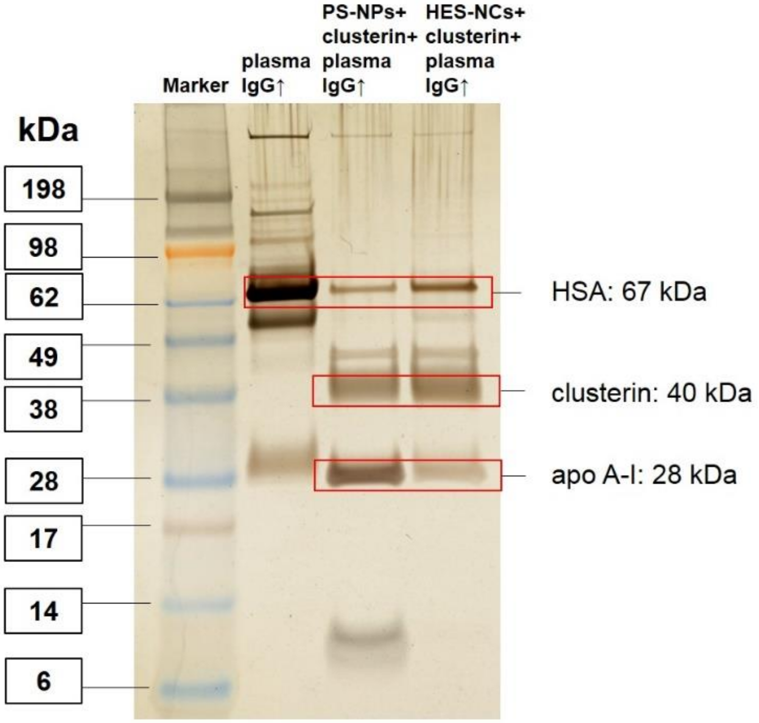

Figure S7: SDS-PAGE gel of the protein coronas of PS-NPs and HES-NCs incubated with IgG-enriched plasma after pre-incubation with clusterin. Pure IgG-enriched plasma is shown as a reference. For staining, a silver staining kit was used according to manufacturer's instruction.

Table S2: NCs that were incubated with normal and IgG-enriched plasma respectively.

abbreviation	material	surfactant	hydrodynamic radii *via* multi-angle-DLS / nm	zeta potential / mV
PS-NPs	polystyrene	Lutensol	52 ± 5	-10
PS-NPs-COOH	polystyrene-COOH	Lutensol	57 ± 6	-29
PS-NPs-NH_2	polystyrene-NH2	Lutensol	51 ± 5	2
PS-NPs-SDS	polystyrene	SDS	53 ± 5	-50
HES-NCs	hydroxyethyl starch	SDS	112 ± 11	-11
SiO2-NCs-CTAC	silica	CTMA-Cl	171 ± 17	-10
SiO2-NCs-LUT	silica	Lutensol	87 ± 8	-10
OVA-NCs	ovalbumin	SDS	272 ± 27	-24
OVA-NCs-PEG	ovalbumin - PEGylated	SDS	261 ± 26	-31
PS-NPs-PMEP	polystyrene	poly(methyl ethylene phosphate)	52 ± 5	-48

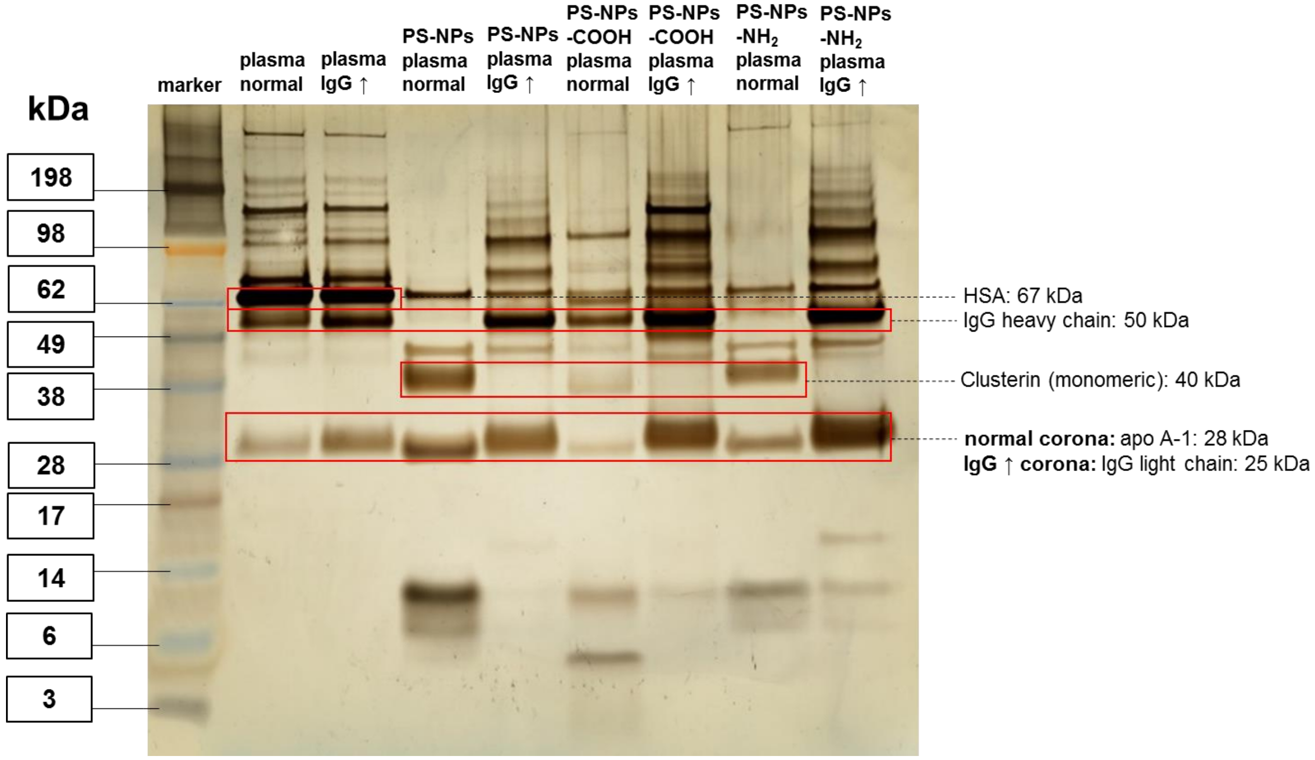

Figure S8: SDS-PAGE gel of the protein coronas of different NCs incubated with normal and IgG-enriched plasma. Pure normal and IgG-enriched plasma is shown as a reference. For staining, a silver staining kit was used according to manufacturer's instruction.

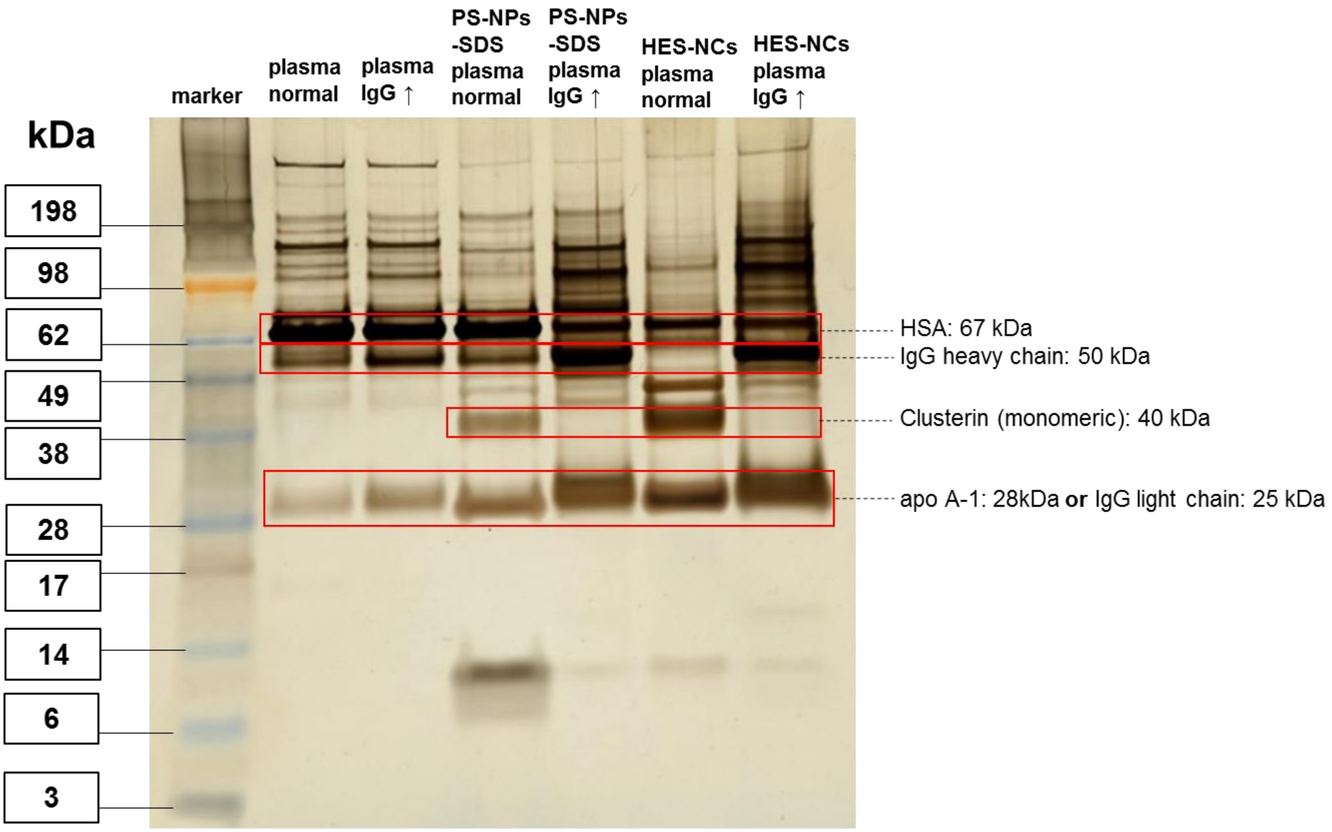

Figure S9: SDS-PAGE gel of the protein coronas of different NCs incubated with normal and IgG-enriched plasma. Pure normal and IgG-enriched plasma is shown as a reference. For staining, a silver staining kit was used according to manufacturer's instruction.

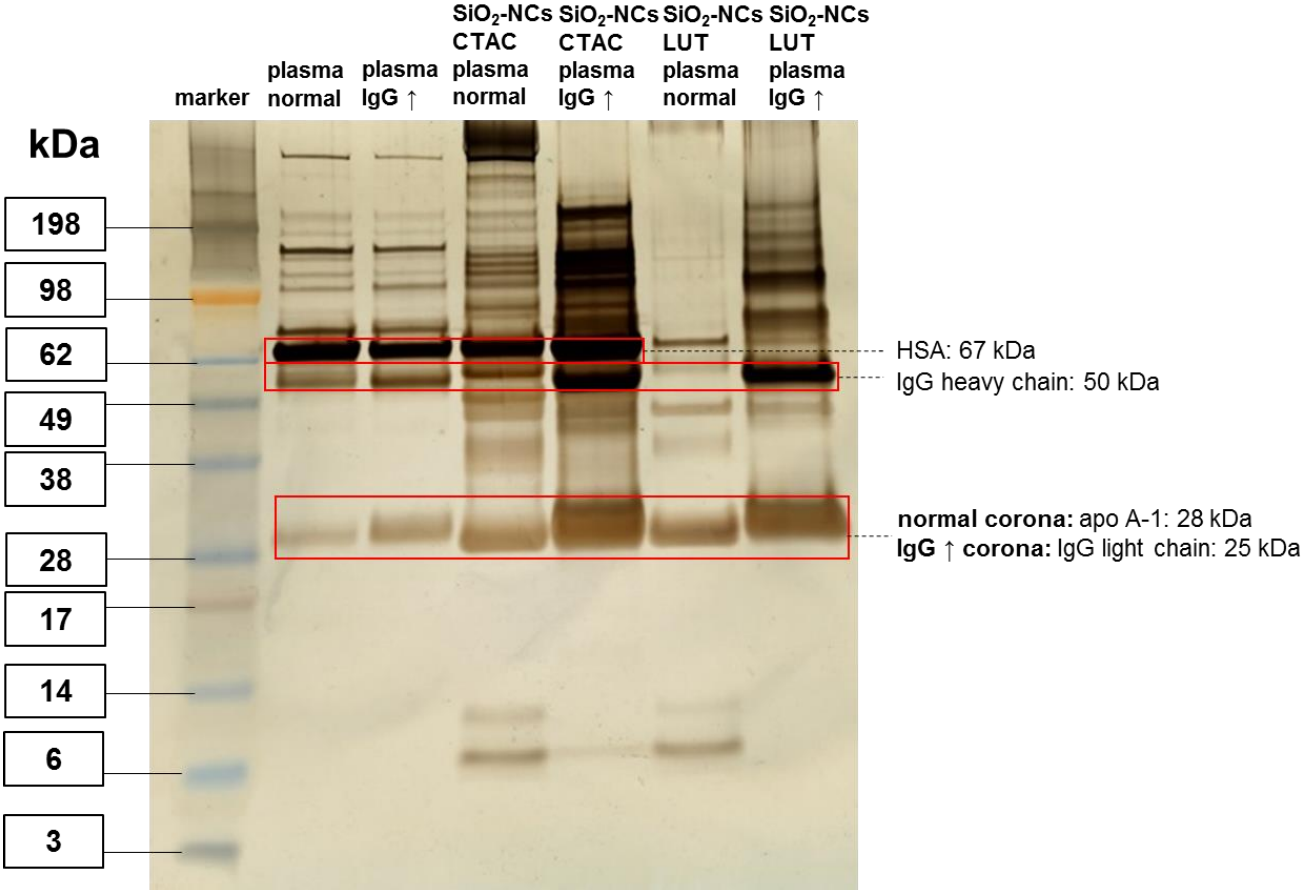

Figure S10: SDS-PAGE gel of the protein coronas of different NCs incubated with normal and IgG-enriched plasma. Pure normal and IgG-enriched plasma is shown as a reference. For staining, a silver staining kit was used according to manufacturer's instruction.

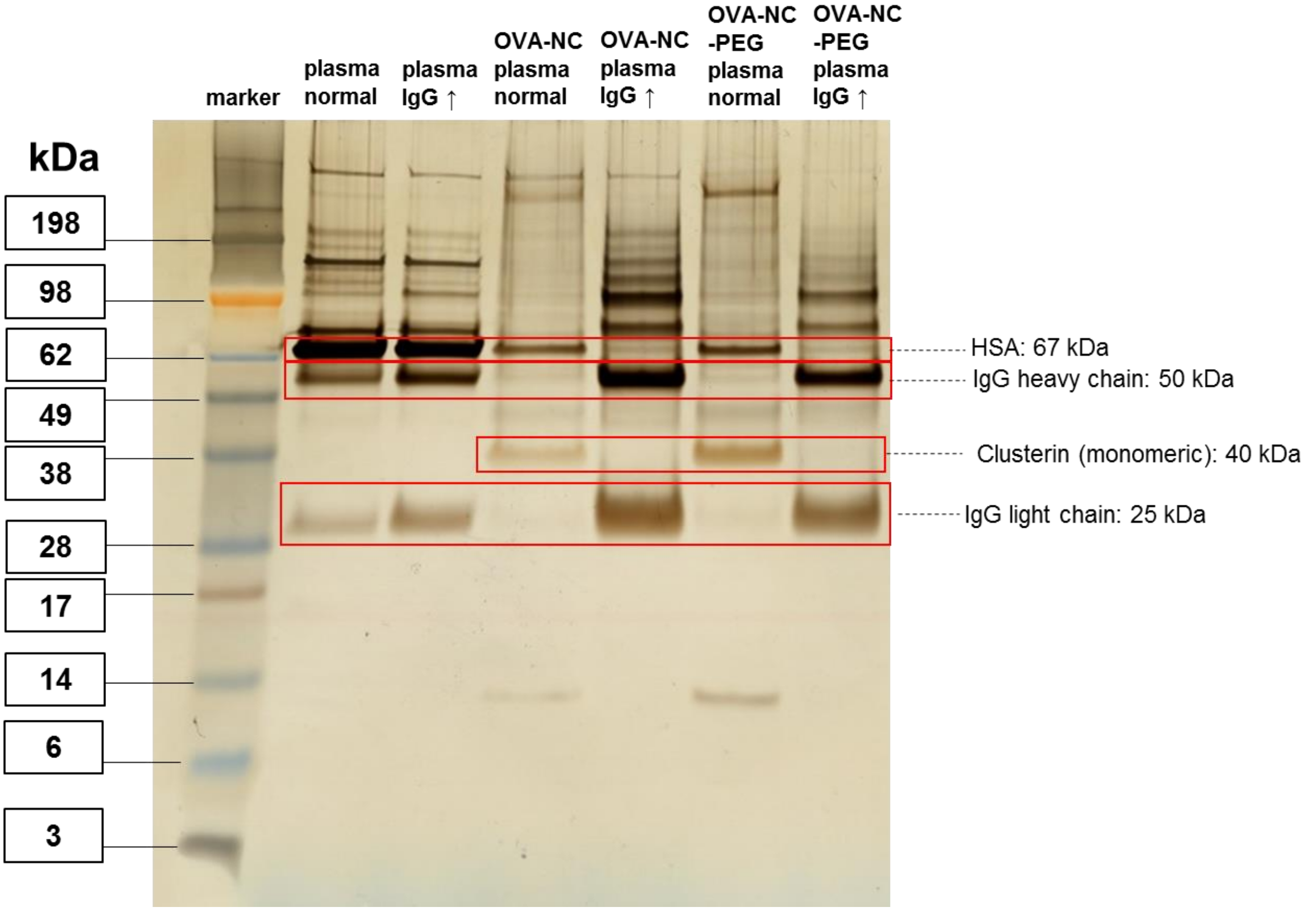

Figure S11: SDS-PAGE gel of the protein coronas of different NCs incubated with normal and IgG-enriched plasma. Pure normal and IgG-enriched plasma is shown as a reference. For staining, a silver staining kit was used according to manufacturer's instruction.

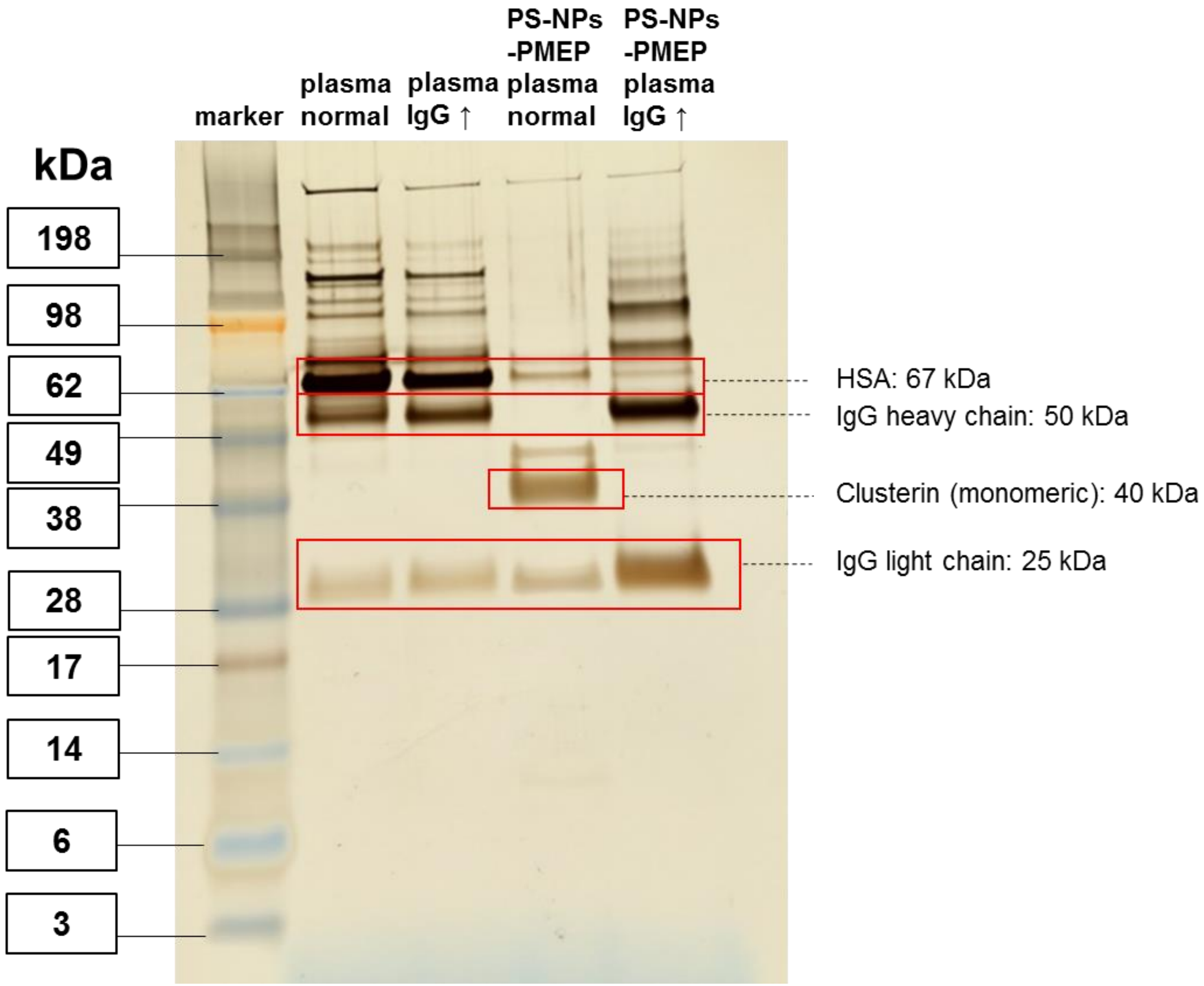

Figure S12: SDS-PAGE gel of the protein coronas of different NCs incubated with normal and IgG-enriched plasma. Pure normal and IgG-enriched plasma is shown as a reference. For staining, a silver staining kit was used according to manufacturer's instruction.

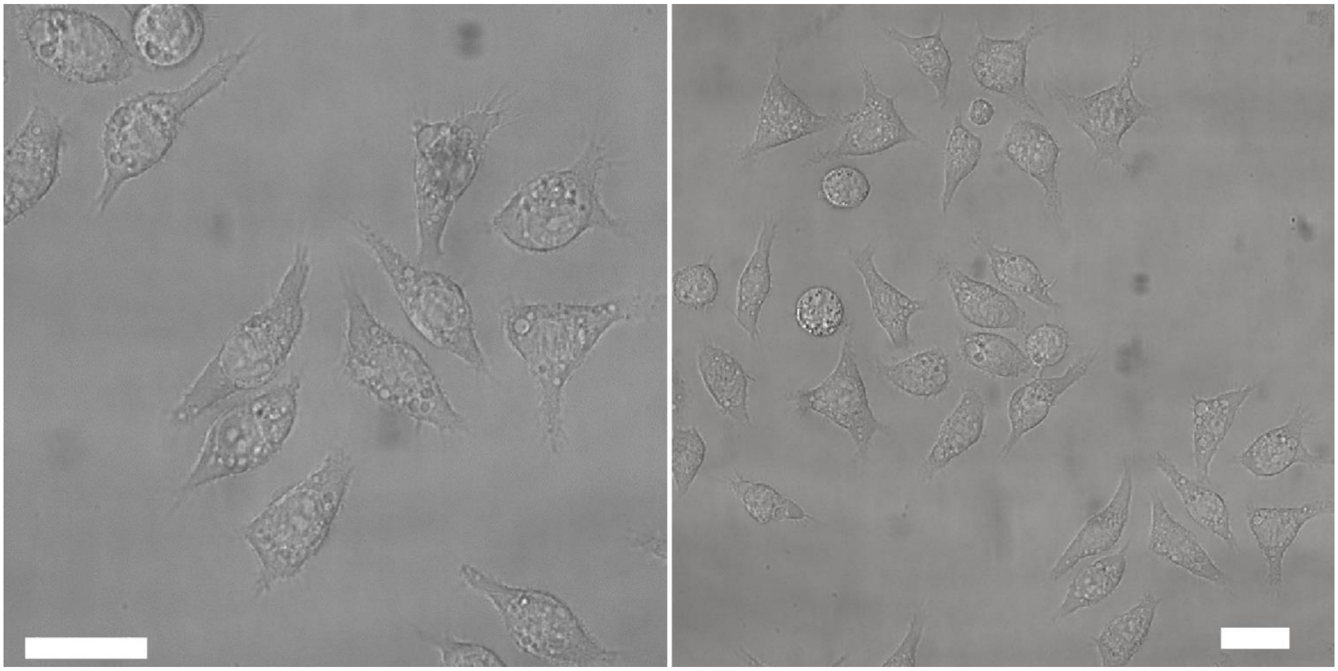

Figure S13: Additional cLSM pictures of RAW 264.7 cells without nanocarriers (negative control). The scale bar corresponds to a length of 20 µm.

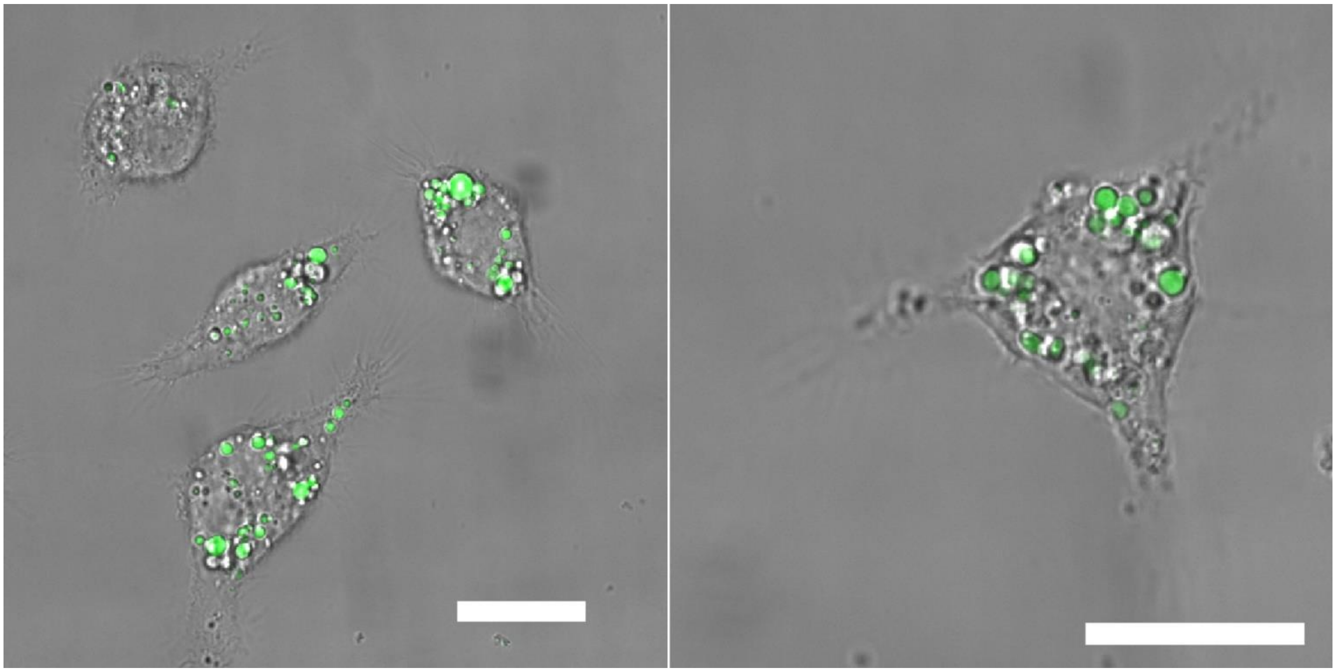

Figure S14: Additional cLSM pictures of PS-NPs in RAW 264.7 cells. Exemplary cLSM pictures were chosen to distinguish cellular uptake from cell membrane decoration. The scale bar corresponds to a length of 20 µm.

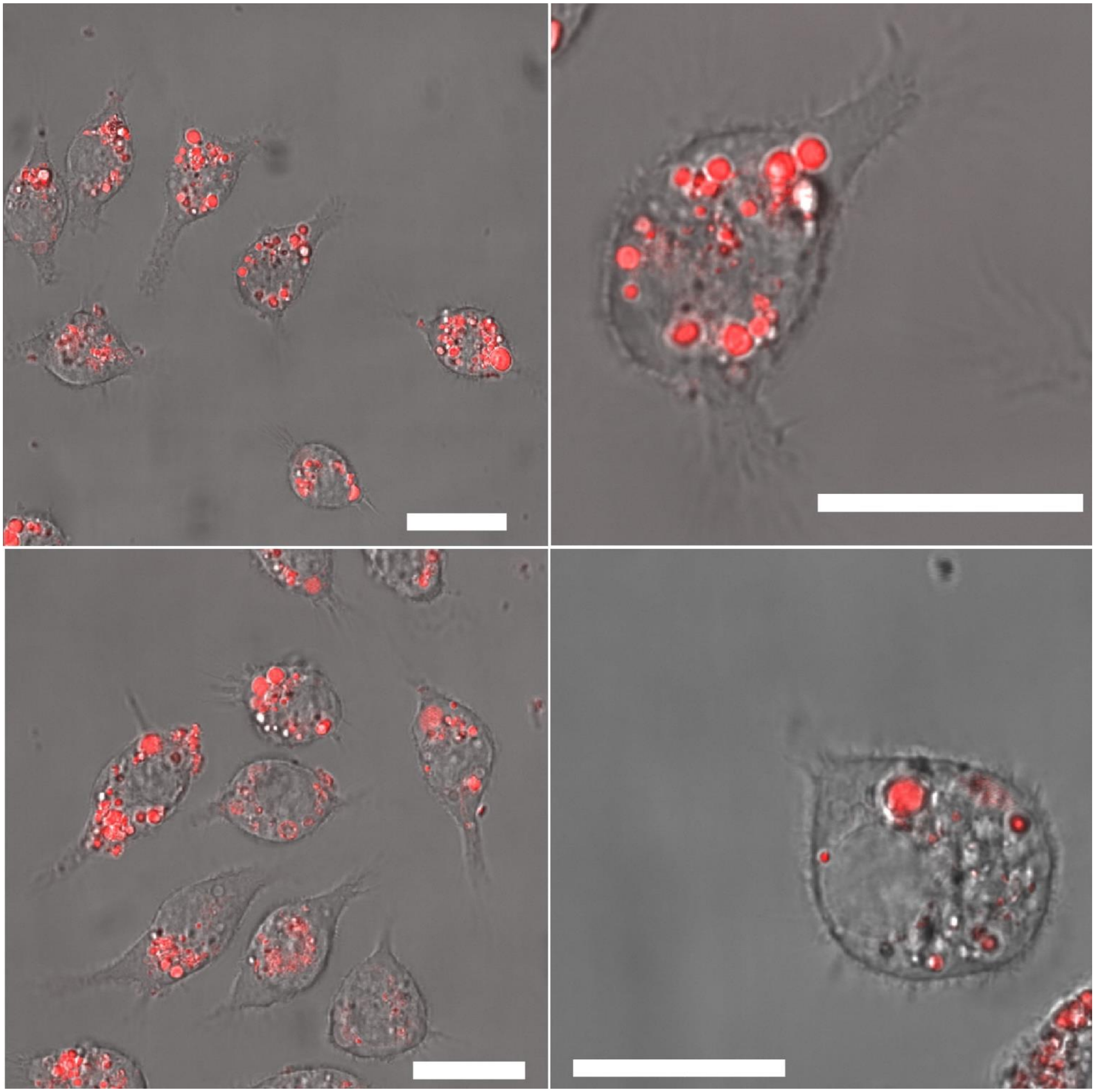

Figure S15: Additional cLSM pictures of HES-NCS in RAW 264.7 cells. Exemplary cLSM pictures were chosen to distinguish cellular uptake from cell membrane decoration. The scale bar corresponds to a length of 20 µm.

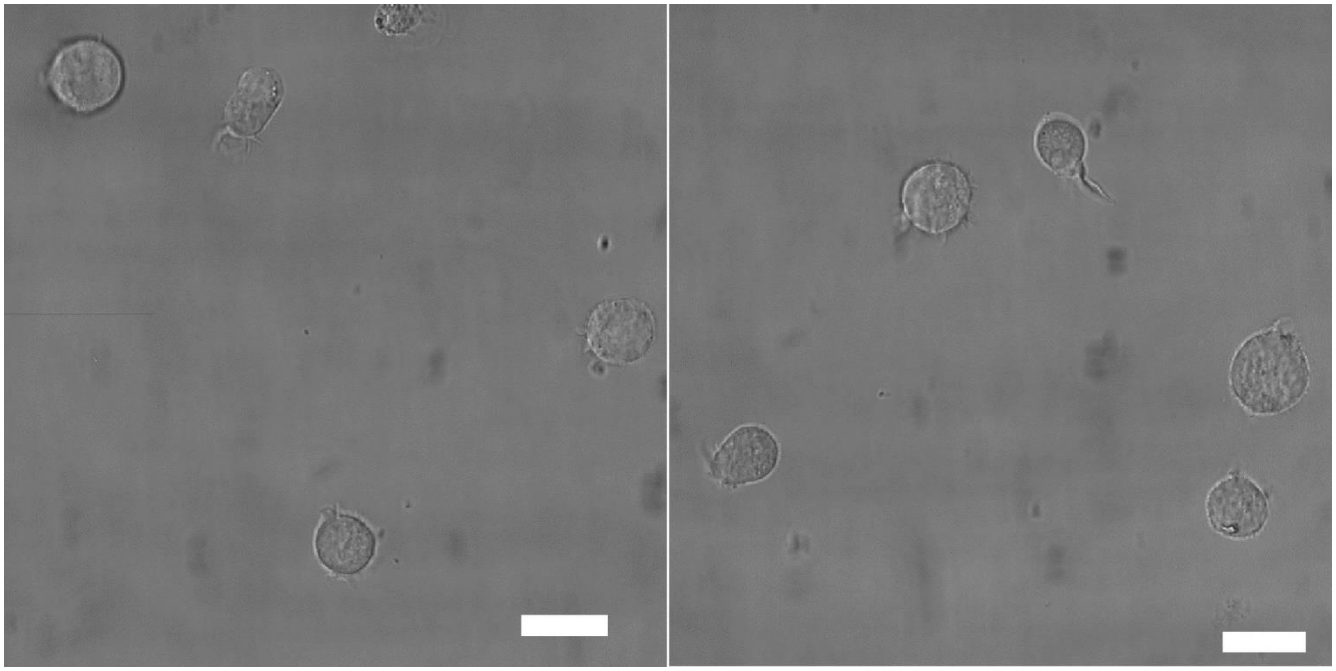

Figure S16: Additional cLSM pictures of THP-1 cells without nanocarriers (negative control). The scale bar corresponds to a length of 20 µm.

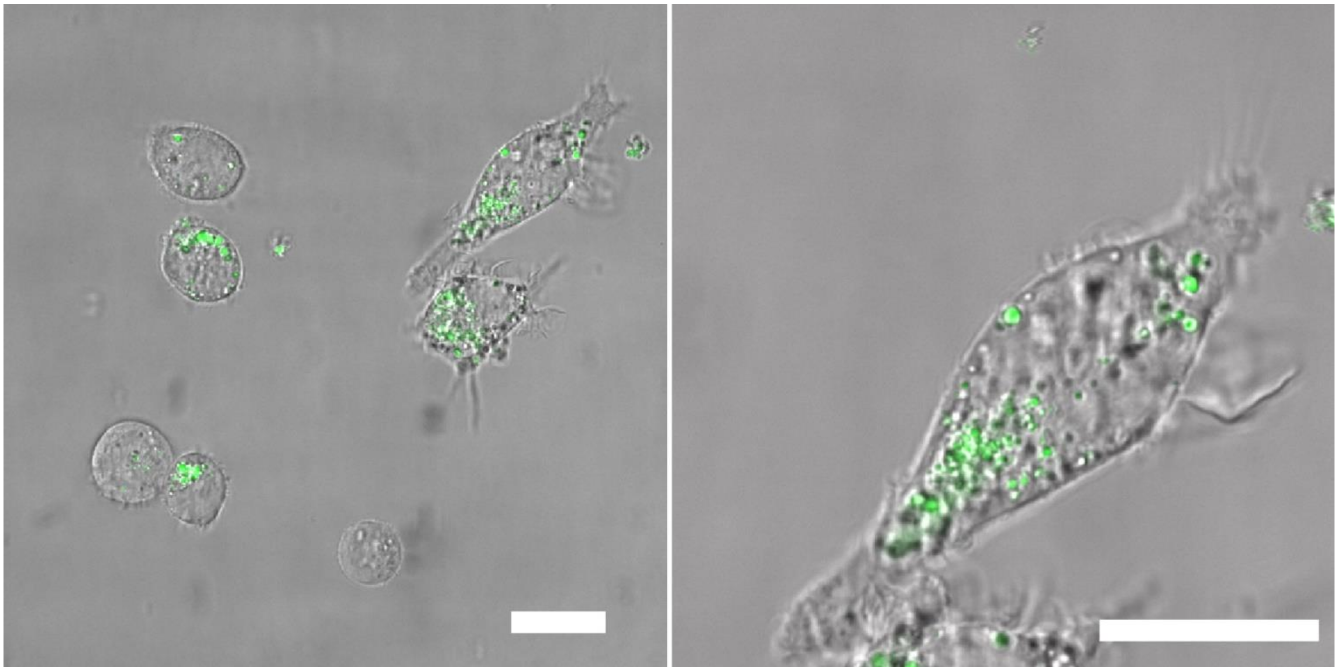

Figure S17: Additional cLSM pictures of PS-NPs in THP-1 cells. Exemplary cLSM pictures were chosen to distinguish cellular uptake from cell membrane decoration. The scale bar corresponds to a length of 20 µm.

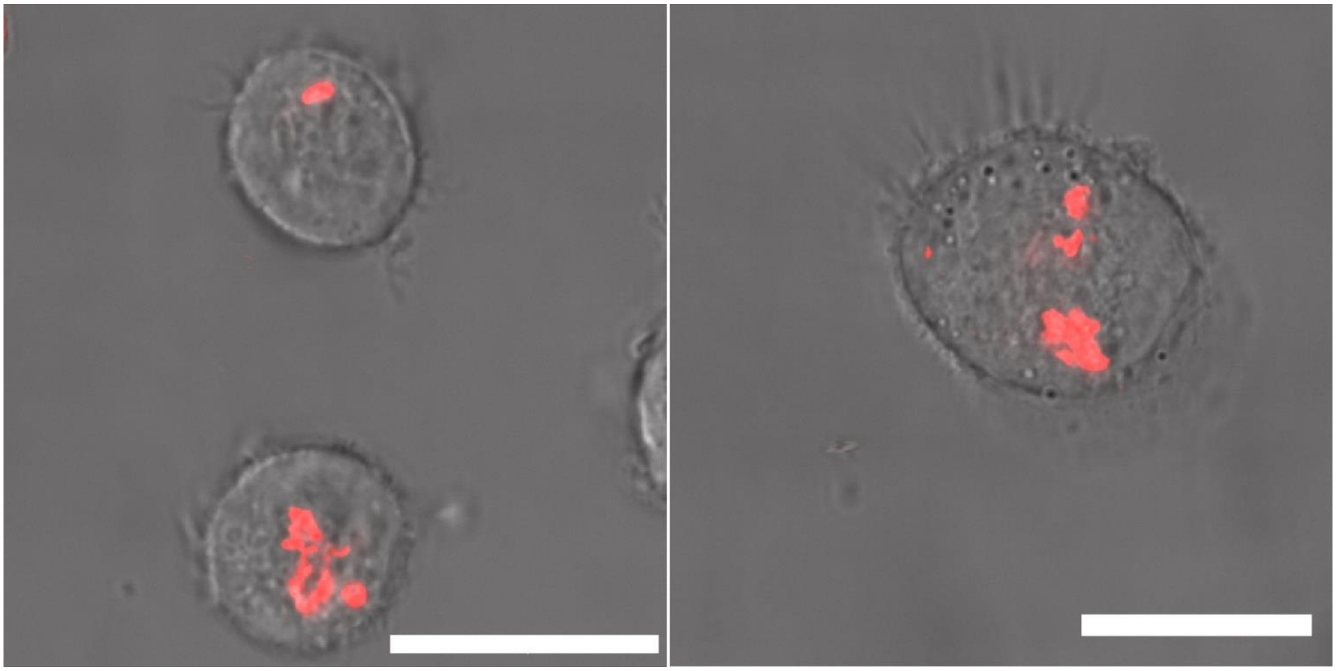

Figure S18: Additional cLSM pictures of HES-NCs in THP-1 cells. Exemplary cLSM pictures were chosen to distinguish cellular uptake from cell membrane decoration. The scale bar corresponds to a length of 20 µm.

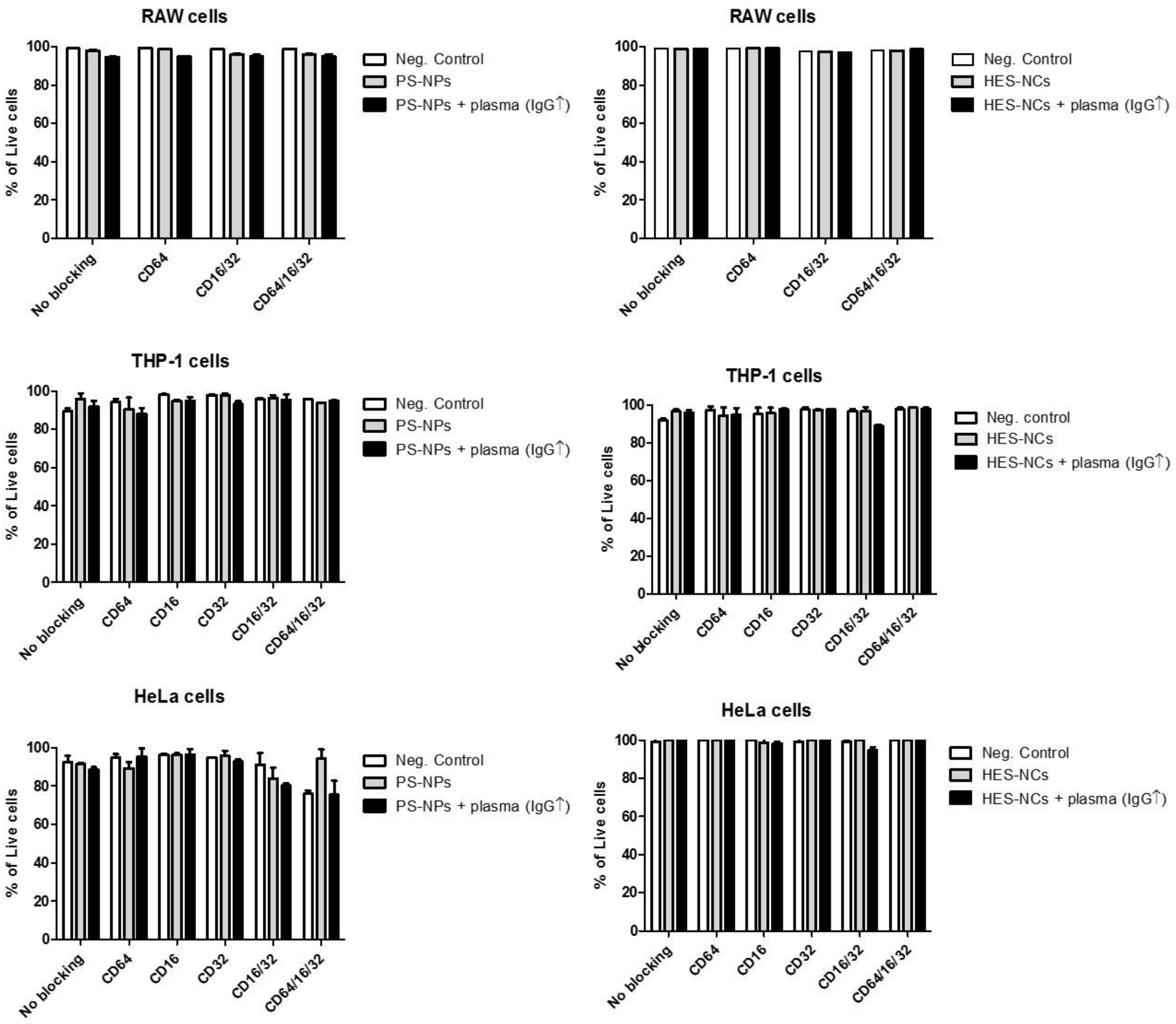

Figure S19: Cell viability tests via Zombie Aqua viability kits. Percentage of live cells for blocking experiments of PS-NPs (left) and HES-NCs (right) before and after incubation with IgG enriched plasma. RAW 264.7, THP-1 and HeLa cells were used as cell lines. Values are mean values with standard deviation of three biological replicates.

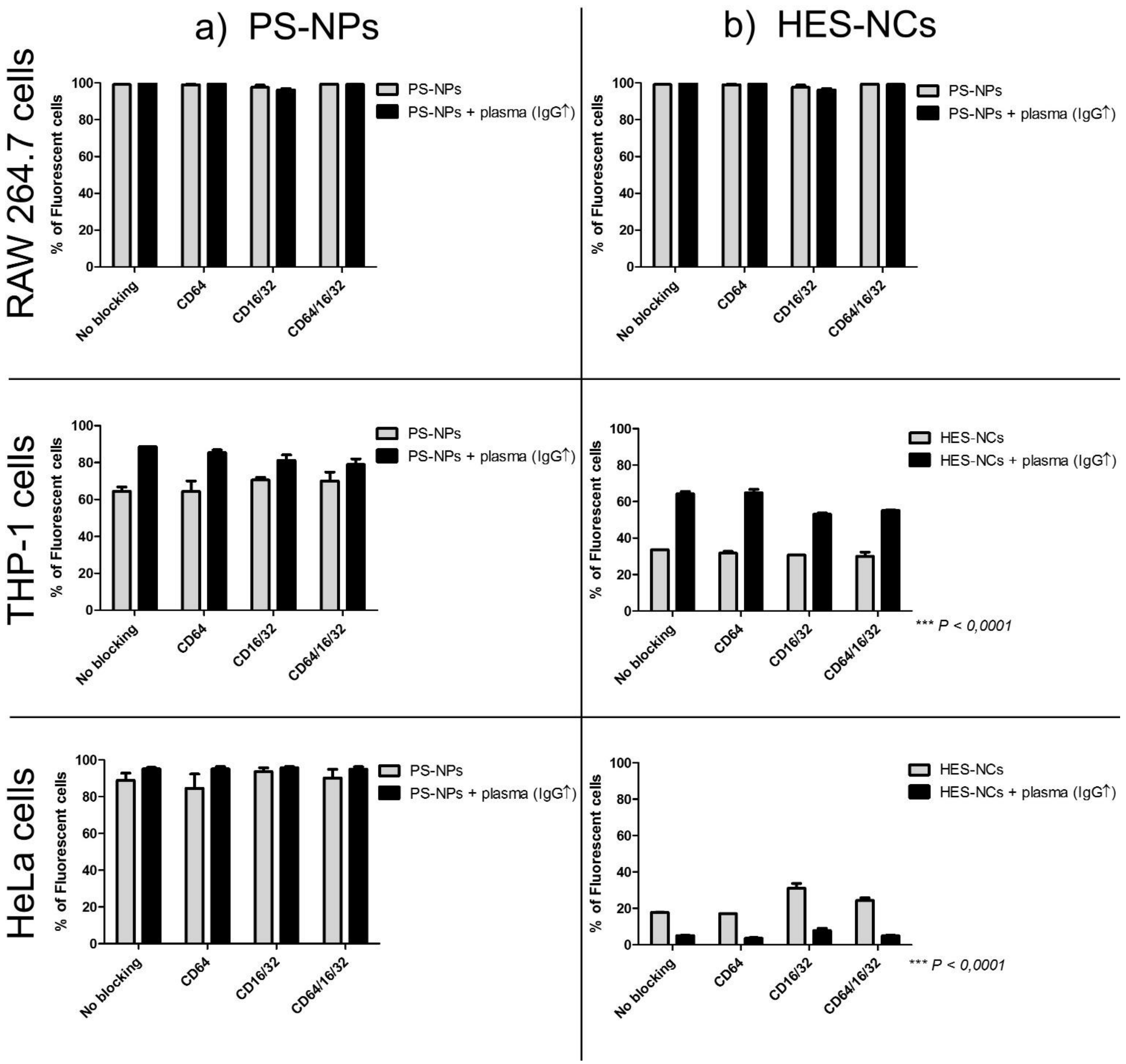

Figure S20: Percentage of fluorescent cells for Fc blocking experiments of a) PS-NPs and b) HES-NCs before and after incubation with IgG enriched plasma. RAW 264.7, THP-1 and HeLa cells were used as cell lines. Values are mean values with standard deviation of three biological replicates. The ANOVA two-way test was used as statistical analysis. P-values describe the interaction between NCs (without protein corona or with corona from IgG-enriched plasma) and blocked/unblocked receptors. Where no significance was observed, p-values are not shown.

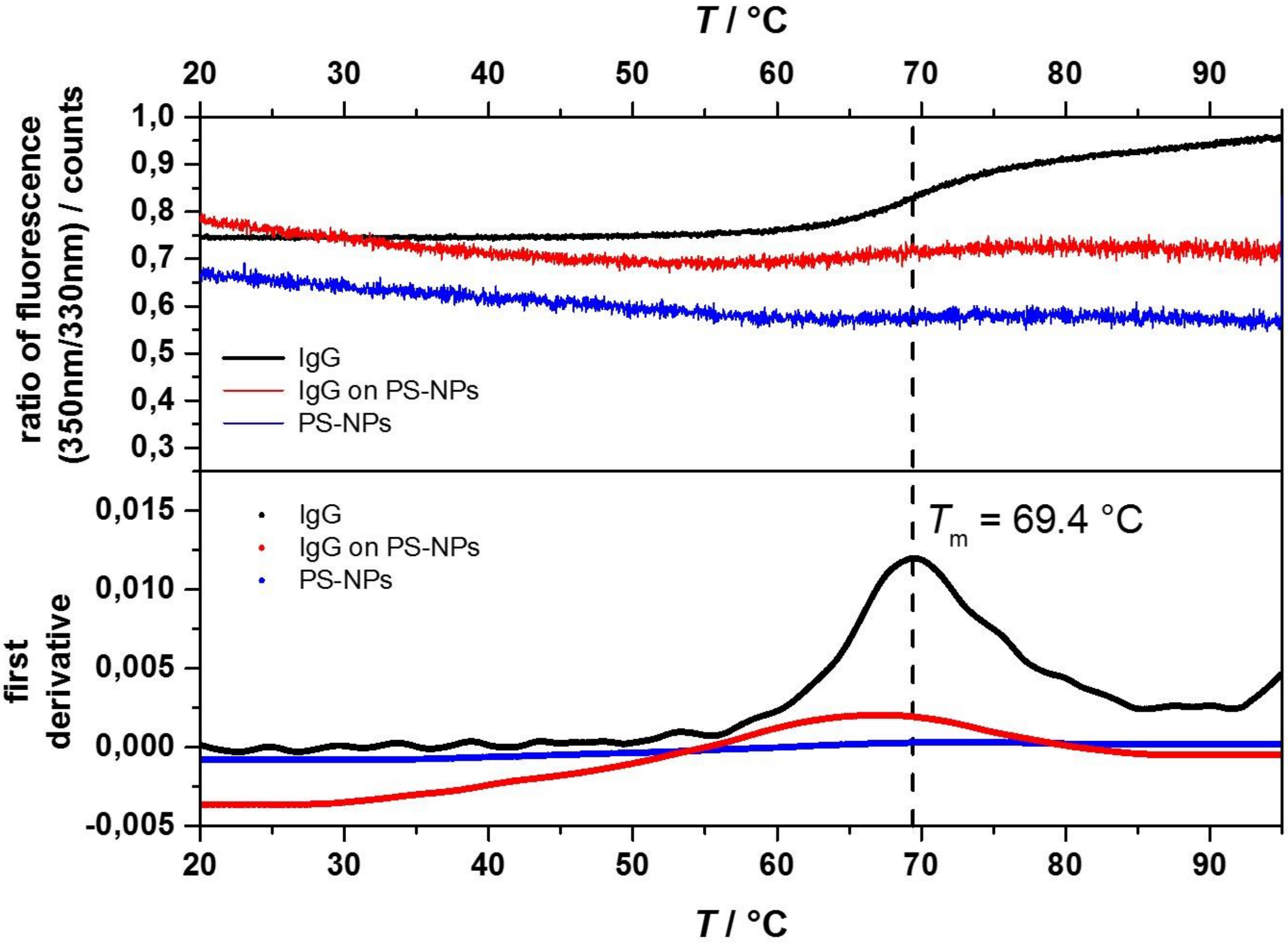

Figure S21: DSF of native IgG (black line —, positive control), pure PS-NPs (blue line —, negative control) and IgG on PS-NPs (red line —) showing the protein unfolding (heating): 350 / 330 nm ratio of fluorescence together with the first derivative. The melting point of native IgG was observed at T_m = 69.4 °C. For pure PS-NPs no change in fluorescence was observed. IgG on PS-NPs after incubation and washing steps did not exhibit any melting transition. This indicates that the protein was already in its denatured form on the surface of the PS-NPs and no native protein remains after the corona preparation.

Supplemental References

1. Lewis, E.A. and Murphy, K.P., *Isothermal Titration Calorimetry*, in *Protein-Ligand Interactions*, G.U. Nienhaus, Editor. 2005, Humana Press Inc: Totowa, New Jersey 07512.
2. Freire, E., Mayorga, O.L., and Straume, M., Isothermal Titration Calorimetry. *Analytical Chemistry*, **1990**, *62*(18), A950-A959.
3. Provencher, S.W., CONTIN: A general purpose constrained regularization program for inverting noisy linear algebraic and integral equations. *Comput. Phys. Commun.*, **1982**, *27*(3), 229-242.
